# Reverse engineering of feedforward cortical-Hippocampal microcircuits for modelling neural network function and dysfunction

**DOI:** 10.1038/s41598-024-77157-4

**Published:** 2024-10-29

**Authors:** Katrine Sjaastad Hanssen, Nicolai Winter-Hjelm, Salome Nora Niethammer, Asgeir Kobro-Flatmoen, Menno P. Witter, Axel Sandvig, Ioanna Sandvig

**Affiliations:** 1 Department of Neuromedicine and Movement Science, Faculty of Medicine and Health Sciences, Norwegian University of Science and Technology (NTNU), Trondheim, Norway; 2 Kavli Institute for Systems Neuroscience, Centre for Neural Computation, Egil and Pauline Braathen and Fred Kavli Centre for Cortical Microcircuits, Norwegian University of Science and Technology (NTNU), Trondheim, Norway; 3 Division of Neuronal Cell Biology, Center for Brain Research, Medical University of Vienna, Vienna, Austria; 4 K.G. Jebsen Centre for Alzheimer’s Disease, Faculty of Medicine and Health Sciences, Norwegian University of Science and Technology (NTNU), Trondheim, Norway; 5 Department of Neurology and Clinical Neurophysiology, St Olav’s University Hospital, Trondheim, Norway

**Keywords:** Microfluidic device, Electrophysiology, MEA, Adult neural networks, Alzheimer’s model, In vitro, Biomedical engineering, Neural circuits, Tissue engineering, Neurological disorders

## Abstract

Engineered biological neural networks are indispensable models for investigation of neural function and dysfunction from the subcellular to the network level. Notably, advanced neuroengineering approaches are of significant interest for their potential to replicate the topological and functional organization of brain networks. In this study, we reverse engineered feedforward neural networks of primary cortical and hippocampal neurons, using a custom-designed multinodal microfluidic device with Tesla valve inspired microtunnels. By interfacing this device with nanoporous microelectrodes, we show that the reverse engineered multinodal neural networks exhibit capacity for both segregated and integrated functional activity, mimicking brain network dynamics. To advocate the broader applicability of our model system, we induced localized perturbations with amyloid beta to study the impact of pathology on network functionality. Additionally, we demonstrate long-term culturing of subregion- and layer specific neurons extracted from the entorhinal cortex and hippocampus of adult Alzheimer’s-model mice and rats. Our results thus highlight the potential of our approach for reverse engineering of anatomically relevant multinodal neural networks to study dynamic structure-function relationships in both healthy and pathological conditions.

## Introduction

Engineered biological neural networks facilitate the study of dynamic properties of network structure and function at the microscale (i.e., subcellular and cellular level) and mesoscale (i.e., network level), in highly controllable experimental conditions. There is compelling evidence that neurons *in vitro* maintain their inherent self-organizing properties and over time form neural networks with complex structure and function, thus recapitulating fundamental behaviour of brain networks^1–4^. As such, engineered neural networks offer a complementary approach to *in vivo* studies for investigations of neural structure and function from the subcellular to the network level. In recent years, several methodological and technological developments have enabled advanced neural network modelling *in vitro* using microfluidic devices to engineer multinodal neural networks^5,6^. In these devices, neural populations are confined to distinct chambers, also referred to as nodes, connected by microtunnels that are only permissible for neurites^5,7,8^. This design allows for the segregation of different neuronal populations across the microfluidic chambers while enabling communication between them through the microtunnels. These devices can also be interfaced with microelectrode arrays, allowing for detailed electrophysiological recordings^9,10^. This integration enables the monitoring of both structural and functional network alterations with high spatio-temporal resolution^11^. However, most studies to date are typically limited to using two-nodal microfluidic devices^7,12^. Thus, the potential of such neuroengineering approaches to recapitulate anatomically relevant network configurations is still underutilized. Additionally, the relevant models tend to be limited in terms of their reproducibility, scalability, and adaptability to support multinodal, directional topological configurations resembling *in vivo* neural assemblies.

A key determinant for establishing anatomically relevant neural network configurations *in vitro* is facilitation and control of multinodality. Neural assemblies in the brain are structured into highly specialized functional regions connected through precise unidirectional axonal pathways, aiding feedforward and/or feedback signal propagation between the nodes^13^. During neural development in the brain, directional projection sequences are established by finely tuned spatiotemporally regulated molecular and chemical cues determining axon growth and guidance^14–16^. Since such gradients are absent in an *in vitro* microenvironment, alternative approaches are required to facilitate establishment of directional projections between the neural nodes. Several recent studies, including studies from our group, have demonstrated that directional axonal outgrowth between interconnected nodes can be achieved and controlled by embedding geometrical constraints within the internodal microtunnel architecture in microfluidic interfaces^17–24^. This approach supports control of connectivity between multiple neural subpopulations with high precision, rendering them particularly suitable for recapitulating anatomically relevant neural networks^25–28^. By interfacing this type of microfluidic devices with microelectrode arrays (MEAs), emerging structure-function dynamics can also be studied electrophysiologically^29^. Previous studies suggest that containment of neural networks into modular configurations can promote the development of a broader repertoire of activity dynamics compared to unstructured networks, akin to complex information processing seen *in vivo*^26,30^. For instance, we and others have shown that structurally coupled neural networks exhibit functional asymmetry, with independently regulated information processing within the interconnected nodes (i.e., intranodal), as well as controlled information flow between them (i.e., internodal)^24,25,31,32^.

A relevant neural network configuration to study by using such interfaces is the highly interconnected entorhinal-hippocampal network, a brain area important for processing and storage of memories^33^. The entorhinal-hippocampal network consists of multiple subregion specific cell types connected through axonal projections. Multinodal microfluidic devices offer an opportunity to reconstruct this network, and to study ongoing structural and functional dynamics in a highly controlled microenvironment. Furthermore, the entorhinal-hippocampal network is particularly vulnerable to Alzheimer’s disease (AD), evidenced by early accumulation of tau pathology^34^, amyloid beta plaque load^35^, altered neuronal activity such as seizure-like hyperactivity^36^, and major neuronal^37,38^ and synaptic loss^39,40^. Thus, this network is of high interest to study at preclinical stages of AD. We and others have demonstrated that adult entorhinal and hippocampal subregion specific cells from AD model mice are able to reconnect in culture^41–43^. We also recently reported a method for long-term (>2 months) culturing and recording of lateral entorhinal cortex layer II (LEC LII) neurons from AD model animals *in vitro*^43^.

In the present study, we demonstrate reverse engineering of anatomically relevant biological neural networks, including networks of layer specific neurons derived from adult AD model animals, using custom-designed microfluidic MEA interfaces. By incorporating Tesla valve inspired microtunnels between nodes, we could structure multinodal neural networks with controllable feedforward connectivity, and record spontaneously evoked and stimulation induced signal propagation between the nodes. This engineering approach supports the longitudinal study of dynamic structure-function relationships in both healthy and diseased neural networks with high temporal and spatial precision. We furthermore demonstrate the applicability of this model system for selectively inducing localized perturbations with human amyloid beta (A$\beta$) fragments, allowing us to study the spread and impact of AD relevant pathology on network functionality. Moreover, we demonstrate long-term culturing of region and layer specific neurons extracted from adult AD-model mice and rats on these platforms in an anatomically relevant configuration. We show that these adult neurons re-form structural connections and develop spontaneous electrophysiological spiking activity after 15 days *in vitro* (DIV). Taken together, our results demonstrate that our reverse engineering approach is highly relevant for advanced preclinical modelling of neural network function and dysfunction, including neurodegeneration.

## Results

### Feedforward cortical-hippocampal networks are readily established in the four-nodal microdevices

**Fig. 1:**
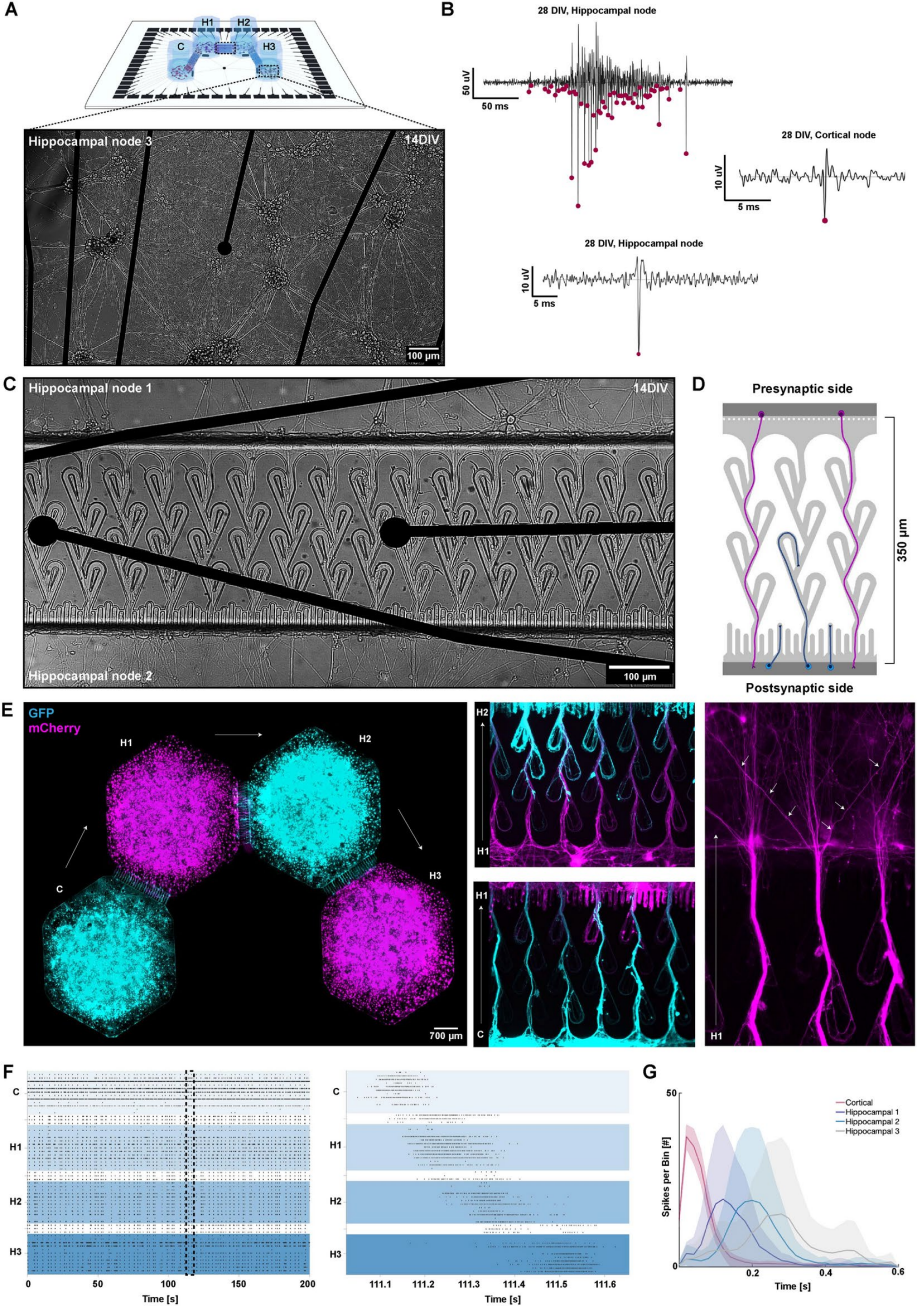
Feedforward connectivity establishment and activity propagation in the four-nodal cortical-hippocampal networks. (**A**) Representative micrograph of a hippocampal node at 14 DIV. (**B**) Voltage traces showing a burst detected within one of the hippocampal nodes at 28 DIV, as well as representative examples of spikes detected within the cortical and hippocampal nodes. Pink specks represent spikes detected by the PTSD algorithm. (**C**) Micrograph showing neurites crossing the Tesla valve inspired microtunnels already after 14 DIV. (**D**) Graphical illustration of how the Tesla valve design promotes neurite outgrowth from the purple cell population across the microtunnels, whereas neurites from the blue cell population get redirected to the chamber from which they originated by the Tesla loops or trapped in the saw-tooths. (**E**) Micrograph of a four-nodal network with neurons expressing either GFP (node C and H2, cyan) or mCherry (nodes H1 and H3, magenta). The images in the middle panel show how neurites from the postsynaptic sides are rerouted back to their node of origin by the Tesla valves. The rightmost panel displays only the magenta channel to illustrate that neurites from the presynaptic side extend through the microchannels and defasciculate into the postsynaptic node. (**F**) Representative raster plot showing the complex network dynamics emerging in the four-nodal networks. A fraction of the network bursts were also clearly spreading from the cortical node throughout all four nodes, as represented in the zoomed-in graph. (**G**) Peristimulus time histograms were created for each network by binning the data in the 300ms following stimulations into 20ms time bins. The graph displays the response of each of the four nodes following repeated (n=60) stimulations of the cortical node for one network at 28 DIV. The thick dark lines (Cortical: pink, Hippocampal 1: purple, Hippocampal 2: blue, Hippocampal 3: gray) represent the mean response of the network for each time bin across the 60 stimulations, while the shaded colors indicate the standard deviation (SD). The stimulations clearly induced feedforward activity propagating throughout all four nodes. DIV: Days *In Vitro*, C: Cortical node, H1-3: Hippocampal node 1-3.

In this study, we reverse engineered multinodal rodent cortical-hippocampal neural networks with controllable unidirectional connectivity using custom-designed microfluidic devices interfaced with microelectrode arrays. Neurons within the six four-nodal devices self-organized into highly complex networks by 14 DIV (Fig. 1A). At this stage, electrophysiological recordings furthermore indicated that spontaneous neural activity had started transitioning from immature tonic firing to synchronous bursting within the nodes, consistent with previous findings (Fig. 1B)^1,3,24,44,45^.

The microfluidic tunnels connecting the individual nodes were designed to promote unidirectional structural connectivity. Tesla valves were included to redirect axons from the postsynaptic side back to their node of origin, while saw-tooths were included on the postsynaptic side to misguide axons growing towards the microtunnel inlets (Fig. 1C and D). To verify unidirectionality, neurons were transduced with AAV viruses for ubiquitous expression of either GFP (nodes 1 and 3) or mCherry (nodes 2 and 4). This verified that the design indeed guided neurites originating in the presynaptic node all the way through the microtunnels, while neurites from the postsynaptic node were rerouted back to their node of origin (Fig. 1E). Although a small number of neurites were observed growing through the microfluidic channels in the unintended direction, this minimal occurrence has previously been shown to be insufficient to cause backpropagation of network bursts^24^.

Functionally, both spontaneously evoked and stimulation induced network bursts were used to assess the capacity of the networks for feedforward activity propagation between the four unidirectionally connected nodes. A fraction of the network bursts initiated in the cortical node, i.e., node 1, could be seen spreading in a feedforward manner throughout all four nodes already at 12 DIV (Fig. 1F). Furthermore, electrical stimulations delivered to the cortical node initiated activity propagating sequentially along the four nodes (Fig. 1G).

### Functional integration increases, while functional segregation is maintained over time in reverse engineered multinodal microcircuits

**Fig. 2:**
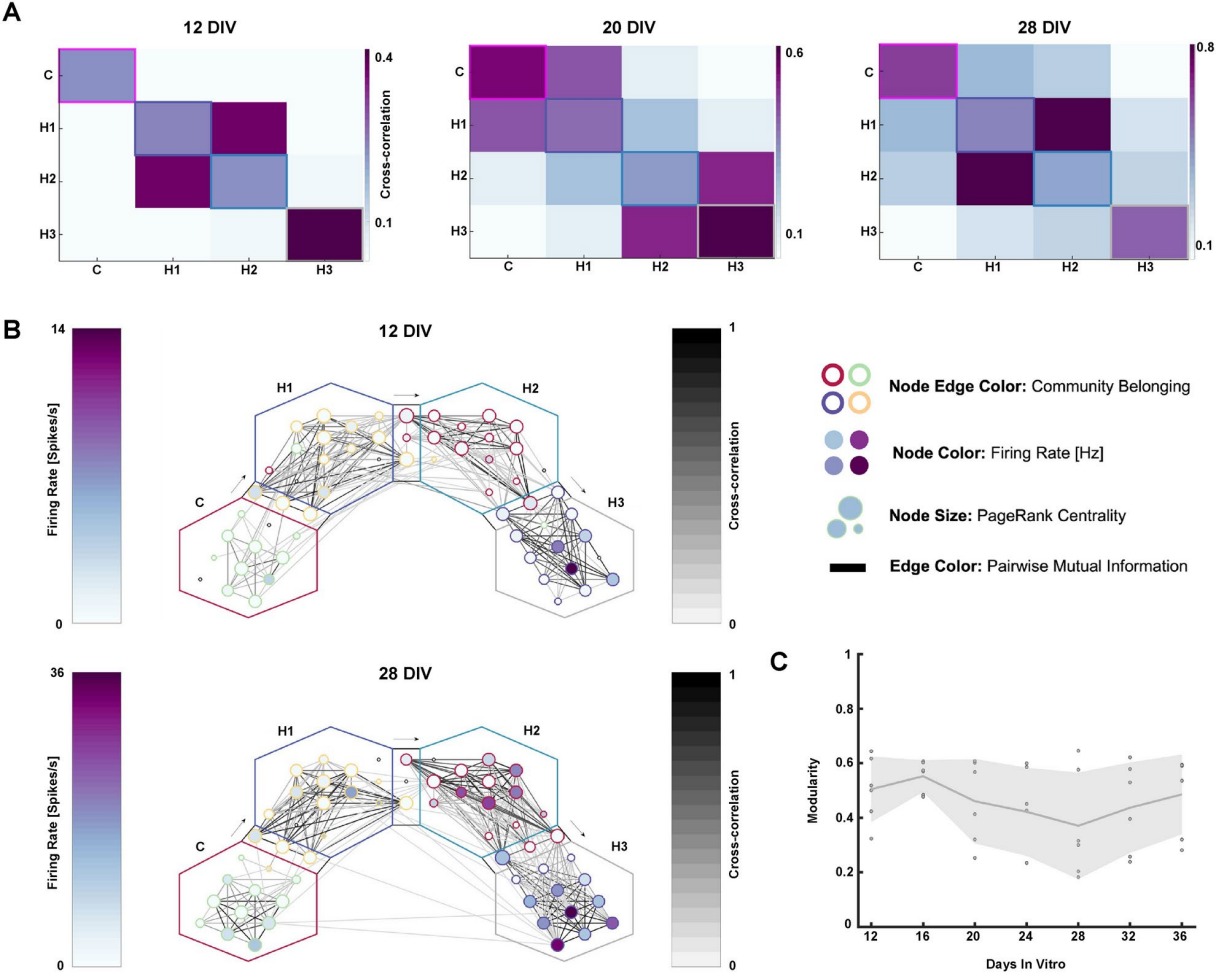
Functional connectivity of the cortical-hippocampal networks. (**A**) Correlation matrices showing the temporal increase in functional connectivity of one representative network over time from 12 to 28 DIV. Note that these matrices are symmetrical and thus do not provide information on the directionality of the functional connections. (**B**) Representative graphs of a single network at 12 and 28 DIV displaying individual electrodes as nodes and their correlation as edges, i.e., lines connecting the nodes. Node color represents firing rate, node size pagerank centrality and the edge color around the nodes which community the node was classified as when the graph was analyzed with the Louvain algorithm. As illustrated, the network can be clearly delineated into distinct communities, with higher intranodal than internodal functional connectivity. These communities are furthermore localized within the distinct nodes of the microfluidic interface. (**C**) Modularity for the four-nodal networks, indicating a retained functional segregation over time. Each point represents a recording from one neural network at a specific developmental time point (DIV), with a total of n = 6 networks. The thick darker grey line indicates the mean maximized modularity value, while the shaded area represents the standard deviation.

The functional connectivity of the networks was evaluated using cross-correlation. Both intranodal and internodal correlation increased over time (Fig. 2A). While some internodal correlation could be observed between nodes already at 12 DIV, integration of all neighboring nodes was not properly established until 20 DIV. At 28 DIV, a higher correlation was also seen between non-neighboring nodes, indicative of a higher network-wide synchronization at this point^24,31,32^. Graph representations of the networks furthermore confirmed that the internodal correlation was clearly strongest between neighboring nodes, while the intranodal correlation on average was higher than the internodal correlation (Fig. 2B).

Furthermore, the entire reverse-engineered cortical-hippocampal networks could be mathematically delineated into four distinct modules using the Louvain algorithm (Fig. 2B). Each of the four detected communities were furthermore physically located within each of the four individual nodes of the microfluidic interface. Despite exhibiting higher internodal correlation with time, the median modularity value stayed close to 0.6 for several networks throughout the experimental period, demonstrating that the networks retained functional segregation of the four nodes (Fig. 2C). The activity recorded by electrodes within the microtunnels also indicated a high pagerank centrality, highlighting their importance for information transfer within the networks (Fig. 2B). These results consequently confirmed that the networks matured into highly integrated multinodal networks with time, while still retaining high capacity for local information processing within the individual nodes.

### Cortical-hippocampal networks exhibit signs of both structural maturation and plasticity at 27 DIV

**Fig. 3:**
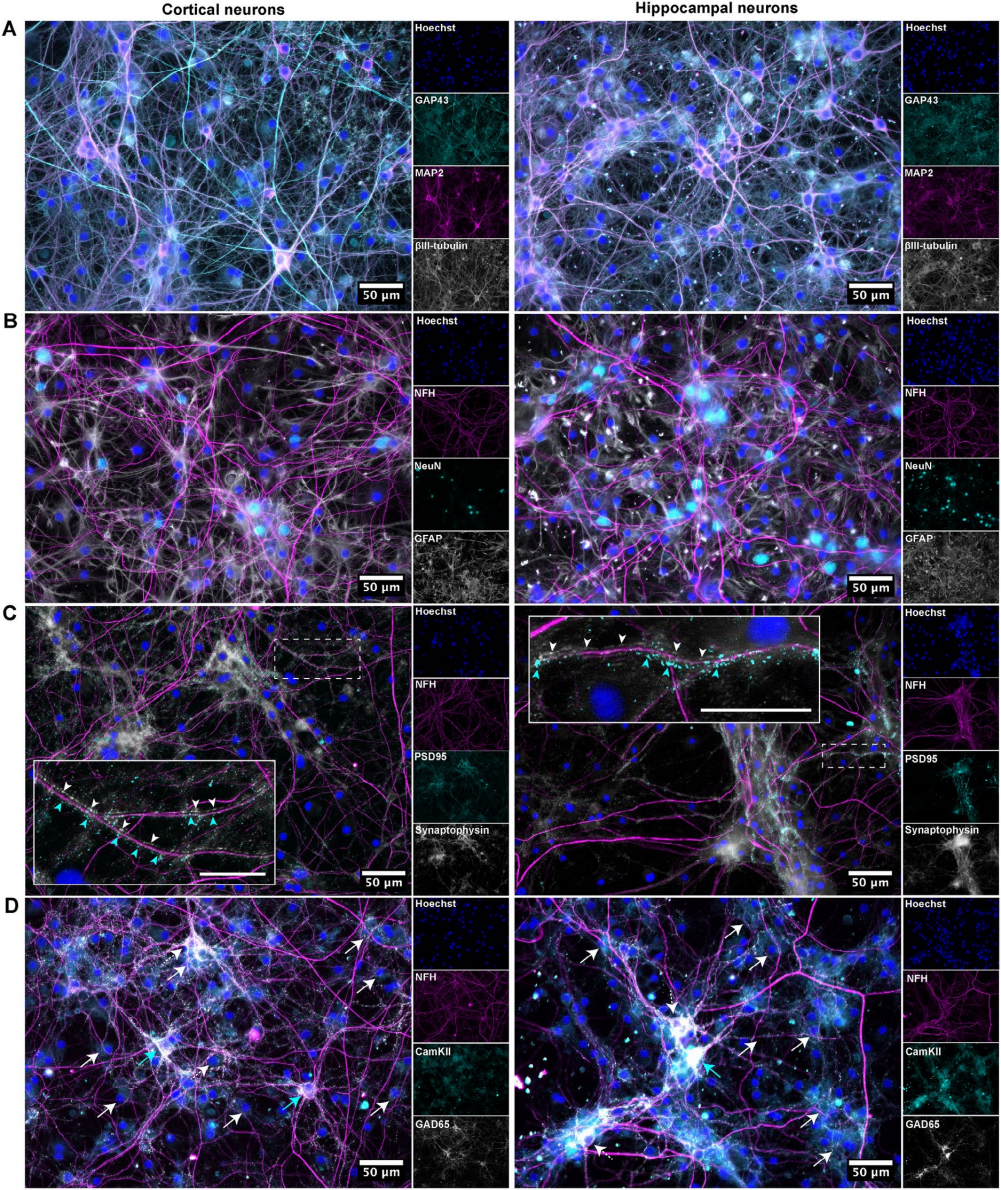
Immunocytochemistry of cortical-hippocampal networks. (**A**) Both cortical and hippocampal neurons self-organized into complex networks, as indicated by the cytoskeletal marker proteins $\beta$3-Tubulin and MAP2. The networks still underwent some level of self-organization and maturation at 27 DIV, as indicated by GAP43, a protein critical for neurodevelopment and growth cone migration. (**B**) The networks had reached a high level of structural maturity at 27 DIV, indicated by the mature markers Neurofilament Heavy (NFH) and NeuN. (**C**) Colocalization of the pre- and postsynaptic markers synaptophysin and PSD95 indicated mature synapses. The enlarged insets show the localization of PSD95 and Synaptophysin clusters, with cyan arrows highlighting PSD95-positive synapses and white arrows indicating Synaptophysin-positive synapses. Scale bar of enlarged insets: ${25}\,{\upmu }{\hbox {m}}$. (**D**) Expression of the glutamatergic and GABAergic markers CaMKII and GAD65 indicated the presence of both excitatory and inhibitory neurons in the networks. White dashed arrows indicate GAD65, white arrows indicate CamKII, and cyan arrows denote their co-localization. A few neurons co-expressed CaMKII and GAD65 indicating the presence of developing neurons in these networks at 27 DIV.

As we saw a high level of functional integration between the nodes beyond 20 DIV, we also evaluated the networks’ structural maturation using immunocytochemistry. To do so, we stained cells in each of the four nodes for both developmental and mature cytoskeletal and nucleic markers at 27 DIV. High levels of Growth-Associated Protein 43 (GAP43) were expressed in both the cortical and hippocampal nodes at 27 DIV (Fig. 3A). This marker was used in combination with the cytoskeletal proteins Microtubule-Associated Protein 2 (MAP2) and $\beta$3-Tubulin, which are expressed from early axogenesis and onwards^46–48^. As GAP43 is critical for neurodevelopment and growth cone migration, the high expression indicates that structural processes were still evolving beyond three weeks *in vitro*^49^. Furthermore, both Neural Nuclear Protein (NeuN) and Neurofilament Heavy (NFH) have been found to be specific for mature neurons, and were used here to assess the maturity of the engineered networks (Fig. 3B)^50,51^. These markers were used in combination with the glial marker GFAP^52^. We found all these proteins to be prominently expressed at 27 DIV, indicating that the networks were reaching structural maturity. This is also consistent with the findings from the electrophysiology, showing highly integrated dynamics between the nodes at this time point (Fig. 2).

Colocalization of the pre- and postsynaptic markers synaptophysin and PSD95 verified the presence of mature synaptic connections (Fig. 3C)^53,54^. Furthermore, distinct expression of CaMKII and GAD65 verified the presence of both glutamatergic and GABAergic neurons, respectively (Fig. 3D)^45,55–57^. Some overlap between GAD65 and CamKII observed in a subset of neurons can be attributed to the co-expression of both GABA and glutamate receptors, which is known to occur in developing neurons^58–60^.

### Amyloid beta oligomerisation remain localized in perturbed nodes, but weaken overall network integration

**Fig. 4:**
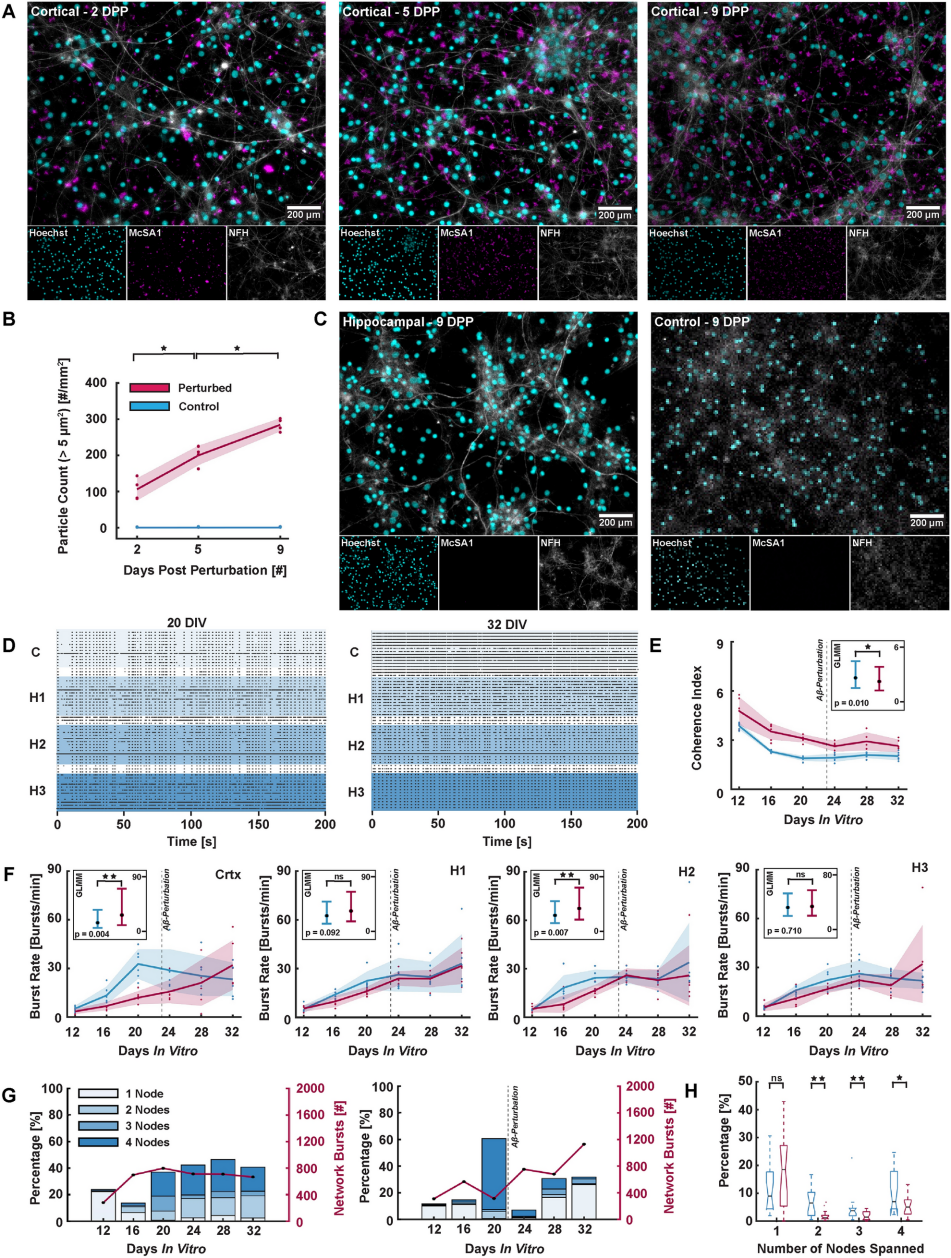
Impact of amyloid beta perturbations on network activity. (**A**) The number of amyloid beta (A$\beta$) oligomers increased over time in the cortical node following perturbations, visualized using the antibody McSA1 staining for insoluble A$\beta$-aggregates. (**B**) The number of particles above ${5}\,{\upmu }{\hbox {m}}$ in diameter increased significantly between 2 - 5 DPP and 5 - 9 DPP (p = 0.0286 for both). Each data point (n = 4 per condition) represents the quantification from one network. The darker lines in the graph show the mean quantification at each measurement time point, while the shaded error bars represent the standard deviation. (**C**) Such aggregates were however not seen in the hippocampal nodes, nor the unperturbed control networks at 2, 5 or 9 days post perturbation (DPP). (**D**) The activity increased over time in particularly the cortical node following perturbations, as illustrated in the raster plots at 20 DIV (prior to perturbation) and 32 DIV (9 days after perturbation) for one representative network. (**E**) The coherence index, indicating network synchrony, was found significantly lower for networks after perturbation (n = 6, 24 - 32 DIV) when compared to the perturbed networks prior to perturbation (n = 6, 12 - 20 DIV) and control networks (n = 6, 12 - 32 DIV) using GLMM estimated group averages with 95 % confidence intervals. (**F**) The burst rate was found significantly higher in the perturbed networks of the cortical node and the second hippocampal node when evaluating the GLMM estimated group averages. The difference between the perturbed and unperturbed networks of the other two hippocampal nodes were however found to be non-significant. Each measurement point represents the median burst rate across all electrodes in the highlighted node (Crtx, H1, H2 and H3) within a single network at a specific DIV (totaling n = 6 control networks and n = 6 networks perturbed with amyloid beta deposits). The darker lines indicate the mean values for each condition (control in blue and perturbed in pink) at each DIV, while the shaded error bars represent the standard deviation. (**G**) Histogram showing the percentage of spontaneously evoked network bursts initiated within the cortical population, and the number of nodes they spanned, for one representative control network and one perturbed network. The total number of network bursts initiated at each DIV is shown along the secondary y-axis. A significant change can be seen for the perturbed networks between DIV 20 (prior to perturbation) and DIV 24 (after perturbation). (**H**) The percentage of network bursts initiated in the cortical node spanning 2, 3 and 4 nodes was on average significantly lower for the perturbed networks compared to the controls (p = 0.001, p = 0.005 and p = 0.035, respectively). Each boxplot shows the distribution of network burst propagation percentages across six neural networks for each condition (controls and perturbed networks). The box represents the range within which the middle 50 % of the data falls, while the line inside the box indicates the median value. Whiskers extend to the minimum and maximum values within 1.5 times the interquartile range, and any points outside this range are considered outliers.

To showcase the applicability of the model system for disease modelling, human A$\beta$-fragments were added to the cortical node of six four-nodal networks at 23 DIV. This perturbation led to a significant gradual increase in the number of A$\beta$-oligomers in the cortical node over time (Fig. 4A and B, p = 0.0286). Neither any of the hippocampal nodes, nor the unperturbed control networks, exhibited expression of such A$\beta$-oligomers during this period of time (Fig. 4C).

To examine whether this perturbation affected the electrophysiological behaviour of the networks, we further investigated the change in functional dynamics both within and across nodes of the six four-nodal networks interfaced with MEAs. Both perturbed and control networks transitioned to exhibiting gradually higher firing rates over time. The activity of the cortical node did however appear to transition towards a more hyperactive state after perturbation with A$\beta$ (Fig. 4D). Furthermore, the coherence index, measuring network synchrony, was found to be significantly lower in the perturbed networks when compared to the controls using Generalized Linear Mixed Effect Model (GLMM) estimated group averages (Fig. 4E). It is important to note that while the observed decrease in network synchrony following perturbation is evident, the GLMMs aggregate data over time, which may affect the interpretation. The initially high coherence index in both control and perturbed networks could contribute to this statistical finding. In line with this, the burst rate was found to be significantly higher in the cortical node in the perturbed networks when assessing the GLMM estimated group averages (Fig. 4F). While the burst rate was also found higher in the second hippocampal node, the difference between the perturbed and unperturbed networks was found non-significant in the first and third hippocampal nodes.

The percentage of network bursts initiated in the cortical node and spreading to the next one, two or three nodes increased significantly between 12 and 20 DIV for both controls and perturbed networks (Fig. 4G). In the control networks, at 28 DIV, as much as 24.2 % of the network bursts propagated through all four nodes, compared to less than 2 % at 12 DIV. For comparison 4.5 %, 13.4 % and 4.4 % of the network bursts that were initiated in the cortical node propagated and stopped after the first, second and third node, respectively. This indicated establishment of more mature directional projection sequences between the nodes. The remaining percentages of network bursts were initiated within the second, third or fourth node. Similar trends were found for the other five control networks (Fig. S1). In the perturbed networks, a sharp decline in the number of network bursts spanning beyond the cortical node was observed between 20 DIV (prior to perturbations) and 24 DIV (following perturbations). There were furthermore on average significantly fewer network bursts initiated in the cortical node spanning 2, 3 or 4 nodes in the perturbed networks in the recordings at 24, 28 and 32 DIV compared to the control networks (Fig. 4H, p = 0.001, p = 0.005 and p = 0.035, respectively).

### Adult entorhinal and hippocampal AD neurons re-form structural networks and exhibit electrophysiological activity

**Fig. 5:**
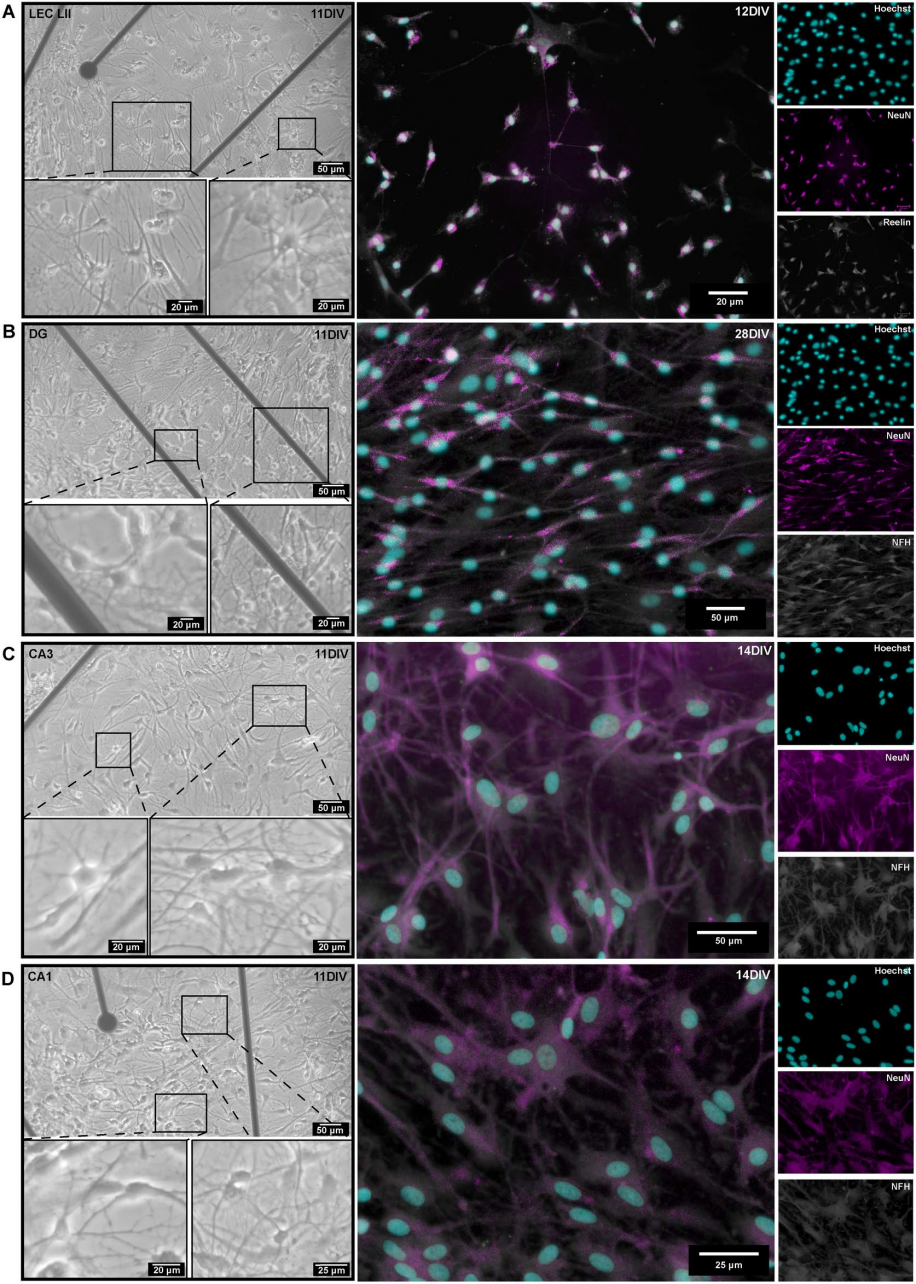
Establishment of adult entorhinal and hippocampal AD neurons on four-nodal microfluidic platforms. (**A**) Left: Phase-contrast images of adult lateral entorhinal cortex layer II (LEC LII) neurons at 11 days *in vitro* (DIV), with enlarged insets from all regions showing neural bodies with extruding neurites. Right: Co-expression of the neural markers NeuN and reelin indicated regional identity at 12 DIV. (**B**) Left: Phase-contrast images of adult dentate gyrus (DG) granular neurons at 11 DIV. Right: Co-expression of neuronal markers NeuN and Neurofilament Heavy (NFH) confirmed neuronal identity at 28 DIV. (**C**) Left: Phase-contrast images of adult Cornu Ammonis 3 (CA3) neurons at 11 DIV. Right: Co-expression of neuronal markers NeuN and Neurofilament Heavy (NFH) at 14 DIV. (**D**) Left: Phase-contrast images of adult Cornu Ammonis 1 (CA1) neurons at 11 DIV. Right: Co-expression of neuronal markers NeuN and Neurofilament Heavy (NFH) at 14 DIV.

To further demonstrate the wider applicability of our model system for reverse engineering anatomically relevant networks for preclinical disease modelling, we used this system to culture neurons extracted from brain regions of interest from adult AD model animals. This method for the dissection and culturing of layer specific entorhinal and hippocampal neurons from AD model rats and mice builds upon our recently published protocol for extraction and culturing of LEC LII neurons from adult AD model APP/PS1 mice^43^. We found that adult neurons from both AD model rats- (Fig. 5) and mice (Fig. S2) were able to re-form structural connections *in vitro*. Furthermore, we found that an astrocytic feeder layer was crucial for attachment of adult entorhinal and hippocampal AD neurons (Fig. S3A). Various combinations of neural media supplements were tested (Supplementary Table S1), and neurites of adult rat neurons showed significantly enhanced outgrowth when adding the growth factor FGF2 (10 ng/mL) after 5 DIV (Fig. S3B and C, all p-values for P3 < 0.01). Immunocytochemistry of the cultured adult neurons showed co-expression of neuronal marker NeuN and reelin in the LEC LII neurons, thus indicating regional identity^61^, and co-expression of NeuN and Neurofilament Heavy for hippocampal DG-gr, CA3-pyr and CA1-pyr. This indicated that the engineered network consisted of the neurons of interest (Fig. 5).

**Fig. 6:**
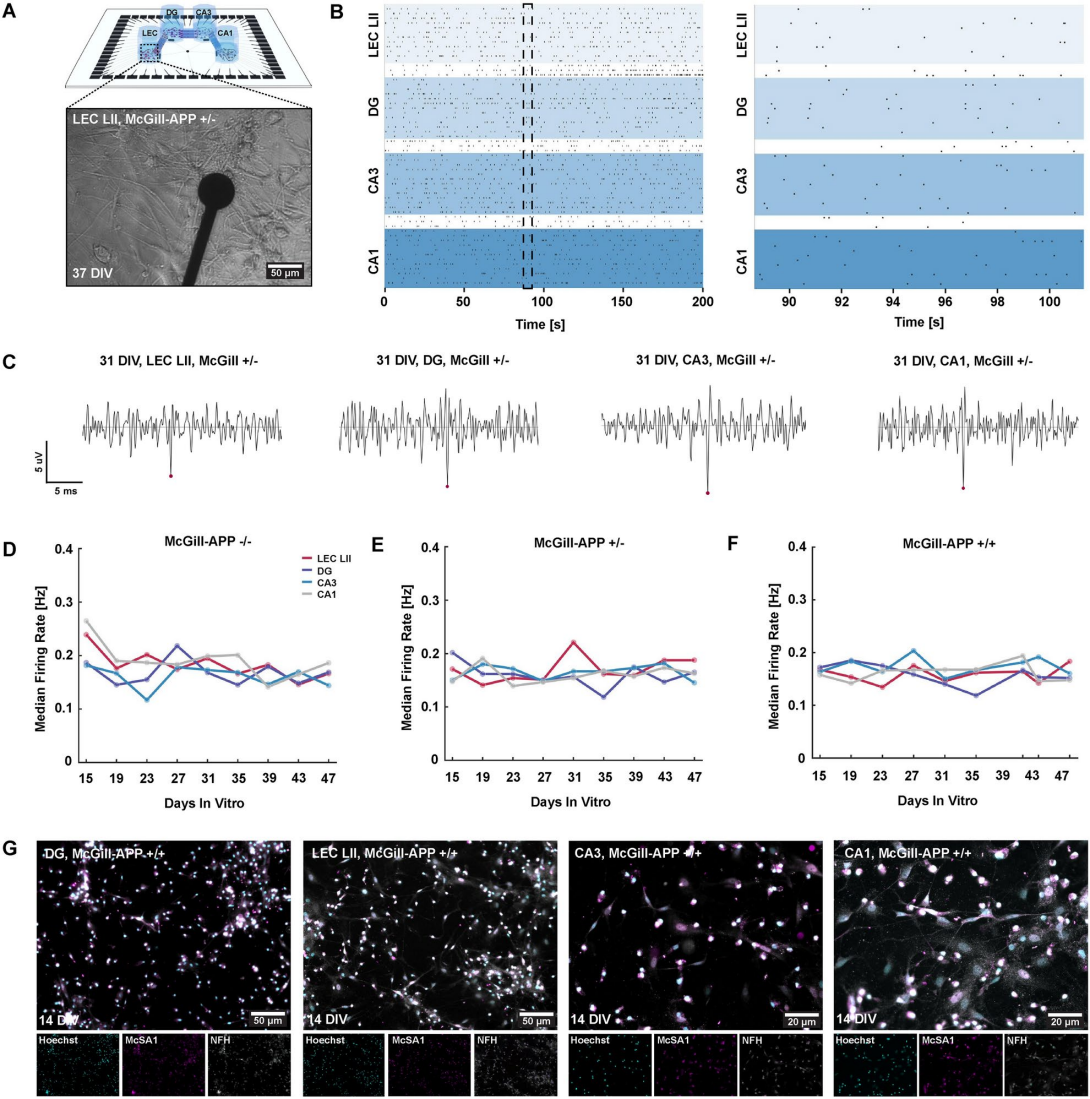
Electrophysiological characteristics of adult entorhinal and hippocampal neural networks from AD-model rats. (**A**) Illustration of a four-nodal microfluidic chip on a microelectrode array with an inset of a phase contrast image of a lateral entorhinal cortex layer II (LEC LII) neural network derived from a McGill-APP rat +/-. (**B**) Representative raster plots at 43 DIV from a network derived from an McGill-Thy1-APP rat model (+/-) showing spontaneous network activity from all four nodes, indicating sparse, desynchronized spikes with no apparent bursting. (**C**) Spike traces showing one representative spike from each subregion, LEC LII (bottom left), DG (top left), CA3 (top right) and CA1 (bottom right) at 31 DIV. (**D**–**F**) Median firing rate (Hz) over time from 15 DIV up to 47 DIV for control networks (McGill-APP -/-) and AD-model rat networks (McGill-APP +/- and McGill-APP +/+). LEC LII; Lateral entorhinal cortex layer II. DG; Dentate gyrus. CA3; Cornu ammonis 3. CA1; Cornu ammonis 3. DIV; Days *in vitro*. McGill-APP -/-: Control rats without the APP K670M671delinsNL and APP V717F mutations. McGill-APP +/-: Heterozygous transgenic rats. McGill-APP +/+: Homozygous transgenic rats. (**G**) Co-expression of markers for amyloid beta (McSA1, magenta), (NFH, grey) in LEC LII and hippocampal DG, CA3 and CA1 neural networks confirm the early accumulation of A$\beta$ in such neurons derived from an adult McGill-APP +/+ rat.

Adult neural networks were furthermore readily established on MEAs (Fig. 6A). From 15 DIV, networks of both AD model rats and mice started exhibiting spontaneously evoked activity (Fig. 6B, 6C and Fig. S4A, respectively). This activity was expressed as sparse, desynchronized spikes with no apparent bursting. Moreover, calculations of the median firing rate showed that neurons from both controls, homozygous and heterozygous McGill-APP rats remained active up to at least 47 days *in vitro* (Fig. 6D–F). Networks of neurons from AD model mice remained active up to 56 DIV (Fig. S4B). ICC furthermore confirmed that the heterozygous and homozygous networks of neurons from McGill-APP rats expressed McSA1, indicative of A$\beta$ aggregates (Fig. 6G).

## Discussion

In this study we have demonstrated reverse engineering of complex multinodal cortical-hippocampal neural networks with controllable unidirectional connectivity. To illustrate the potential of this approach for advanced modelling of neural network function and dysfunction, we selectively induced AD relevant perturbations to healthy neural networks. Additionally, we have demonstrated establishment of layer specific entorhinal-hippocampal networks from neurons dissected from adult AD transgenic rats and mice on our advanced platforms.

For engineered neural networks to reach a mature, computationally efficient state, key design principles such as a modular microarchitecture with directional interconnectivity must be incorporated into the design of the microfluidic MEA interfaces^62–64^. By incorporating Tesla valves in the microtunnels, promoting feedforward connectivity between the interconnected nodes, we showed that the reverse engineered networks organised in a computationally efficient manner. Specifically, by applying a modularity algorithm, we identified distinct communities within the individual nodes of our four-nodal networks. All networks had a high maximized network modularity between 0.3 to 0.6, which for most of the networks remained stable throughout the experimental period. While some networks exhibited decreased modularity beyond 16 DIV, this may be attributed to increased integration between nodes due to the extensive network of microtunnels connecting them. Future experiments varying the number of microtunnels available to the neurites could potentially enhance network modularity further^7^. In our previous study we found the median modularity value to be 0.39 and 0.03 for two-nodal and one-nodal networks, respectively^24^. As the modularity value describes how easily neural networks can be divided into distinct communities, these results illustrate the importance of multinodal interfaces for aiding efficient network organization supporting segregated, yet functionally integrated information processing in different neuronal nodes. Our interfaces were furthermore designed to promote feedforward connectivity between the distinct communities, recapitulating directional information propagation in the brain. To evaluate the efficacy of this design in recapitulating such dynamics, we studied how network bursts initiated in the cortical, i.e. upstream, node spread downstream to the three interconnected hippocampal nodes. These bursts alternated between spanning two, three or four nodes, indicating that the networks had a capacity for gating information, i.e. selectively transmitting only a subset of the information between the distinct communities^24,25,31,32^. Information gating is a fundamental property of neural networks *in vivo*, conferring an ability for segregated and integrated information processing across interconnected nodes. Recapitulation of such complex network behaviour is therefore essential for modelling neural network dynamics in physiological and pathological states, including studying mechanistic effects and functional impact of neurodegenerative disease processes.

One of the major advantages of using the advanced interfaces demonstrated in this study is the ability to capture dynamical structural and functional alterations in the reverse engineered neural networks. By combining fluorescent live imaging with electrophysiology, we could longitudinally monitor the dynamic behaviour of these networks at the micro- and mesoscale. This combined approach can be challenging to implement *in vivo*. An increasing body of literature, including recent studies from our group, has characterized how *in vitro* neural networks develop and mature in healthy, i.e., unperturbed, conditions^1,3,24,44,45^. Such studies provide valuable insights into the intrinsic self-organizing behaviour of engineered neural networks. In this study, we build upon these insights by reverse engineering an anatomically relevant feedforward microcircuit recapitulating key connections in the cortical-hippocampal loop. As such, our novel engineering approach renders the relevant interfaces particularly suitable for a range of biomedical research applications. By selectively inducing neurodegenerative pathology associated with AD in upstream cortical nodes we could monitor network responses across the immediately affected and downstream interconnected nodes. We show that the pathological perturbation caused increased levels of A$\beta$ fragments and hyperactive behaviour in the cortical node, akin to what is seen *in vivo*^65^. With time, developing pathology impacted network integration by reducing the information transmission between the cortical node and the downstream hippocampal nodes. It was also notable that network synchrony appeared to decrease in perturbed networks established from embryonic cells. This observation may reflect developmental changes in network connectivity. In Alzheimer’s disease, a decrease in overall network coherence is often observed alongside localized changes in connectivity, which can lead to increased activity within specific regions^66,67^. Similarly, the decrease in synchrony observed in our perturbed networks might be associated with such localized changes in connectivity, leading to heightened activity within specific regions rather than an increase in overall network activity. These results illustrate that by reverse engineering anatomically relevant neural networks, we can model neurodegenerative pathology and its temporal progression in a controllable microenvironment.

It is worth noting that there were key differences in the developmental timelines of distinct networks in this study. One possible explanation for these differences may lie in the time required for sufficient axonal connections to form across the nodes, facilitating the spread of network bursts. This could also account for the observed variation in the number of network bursts encompassing all nodes. As network bursts begin to propagate between nodes, the receiving node likely adapts, resulting in fewer network bursts originating within that node. In our previous study, we demonstrated that synchronous activity within a postsynaptic node-specifically, the number of network bursts confined to the postsynaptic node without spreading to the presynaptic node-decreased significantly once the nodes were connected, and the postsynaptic node began receiving bursting activity from the presynaptic node^24^. Similarly, in this study, we observed that the total number of network bursts in the perturbed networks decreased significantly coinciding with the point at which a substantial proportion of network bursts began propagating through all four nodes. It is important to note that we set a threshold of 10 % of active electrodes to classify activity as a network burst, meaning that bursting activity originating within a single node, without spreading to others, can still be classified as a network burst if it involves enough electrodes.

As described in the methods section, the control and perturbed networks were derived from two different cell vials, resulting in slight differences in coherence index and burst rate throughout the study. Despite these variations, the overall developmental trends remained consistent, with a decreasing coherence index and an increasing burst rate over time, converging toward 800-1200 network bursts, and peaking in bursts spanning all four nodes between 20 and 24 DIV. Although *in vitro* neural networks often follow similar developmental patterns, factors such as culture interface topography, excitatory/inhibitory balance, and astrocyte support can influence their self-organization and functional profiles. These factors can lead to variations in both structural and functional characteristics, even among neurons from the same batch. As such, when studying network changes over time, particularly in the context of perturbations or disease, trends are often more informative than single time-point differences. A key strength of our study is its longitudinal design, which captures data across multiple time points, providing a more comprehensive understanding of network behavior compared to isolated snapshots. While running experiments in parallel is an alternative approach, it could also introduce additional variability due to the complex study design, which involves multiple protocols and techniques over an extended period. Additionally, this would lead to fewer repeats per condition which could diminish the robustness of observed trends. Nonetheless, despite variations in the data prior to perturbation, our results clearly demonstrate that amyloid beta significantly affects functional connectivity between cortical and hippocampal nodes. This is evident from a shift in the burst rate of the cortical node in control networks, which transitioned from an increasing to a decreasing trend, whereas perturbed networks showed a continuous increase. Furthermore, we show clear disruptions in connectivity between cortical and hippocampal nodes following perturbations. These findings highlight the robustness of this model system for studying longitudinal differences in network developmental trends in response to induced perturbations.

Comparison of healthy networks to networks exhibiting inherent or induced pathological traits can furthermore be used to elucidate key disease mechanisms and therapeutic targets^68,69^. A critical risk factor for several neurodegenerative diseases and disorders, including AD, is age. While the use of embryonic cells can give fundamental insights into the impact of induced pathology, inherent pathology is better modelled using adult cells, retaining the epigenetic mature neuronal profile. By dissecting distinct cellular layers from adult animals, we could establish networks of terminally differentiated non-mitotic neurons from adult rodents with AD pathology. Specifically, we microdissected layer specific entorhinal and hippocampal neurons from adult AD model mice and rats, which are regions known to be affected during early and later stages of AD pathology. We show that the adult neural networks derived from these brain regions self-organized within a week and remained structurally connected over the span of two months. For neurons to remain viable over extended periods, they must form functional connections, as those that do not receive input will eventually undergo synaptic pruning^70^. Such long-term cell viability is essential to model the gradual disease progression of neurodegeneration. Furthermore, we show that the adult neurons displayed desynchronized firing patterns with no apparent bursting, in line with our recent findings from adult mouse entorhinal neural networks^43^. This is also supported by other studies showing that rat cortical and hippocampal neural networks only exhibit sparse, infrequent and brief bursting contrasting the robust, sustained bursting typically seen in embryonic networks when established on MEAs^71^. Similar findings have also been reported from adult hippocampal^72,73^ and entorhinal^74^ slice cultures using patch-clamp recordings, where embryonic cells displayed robust bursting activity, whereas adult cells fired single action potentials. It is also noteworthy that network bursting is a developmental feature of cortical and hippocampal neurons *in vivo*^75^. Here, giant depolarizing potentials (GDPs) are typically present during the early postnatal week(s), before a transition in GABA from depolarizing to hyperpolarizing is seen, which depends on the membrane expression of ion transporters^76^. This underscores the complex interplay between intrinsic network maturation and epigenetic influences. In contrast, adult neurons typically display sparser and more desynchronized activity, which is associated with more efficient neural computations^77,78^. These findings show that networks established from adult neurons exhibit fundamentally different electrophysiological profiles compared to networks matured from an embryonic state *in vitro*. This needs to be taken into consideration when studying neural network behaviour in both healthy and diseased conditions. Although electrical stimulations were not applied to the adult neural networks in this study to avoid introducing additional variability, their use could provide additional valuable insights into the development and functional complexity of adult cells compared to embryonic ones. While further research on the electrophysiological activity of adult neurons is certainly valuable, it is beyond the scope of this manuscript, where our primary goal was to demonstrate the applicability of our model system for studying age-dependent diseases, such as Alzheimer’s. In the present study we reverse engineered entorhinal-hippocampal circuits, however, our approach is widely applicable for reverse engineering of microcircuits from other brain regions as well. Combining multiple interconnected nodes containing brain subregion- and layer specific cells enables the study of progressive disease mechanisms affecting interconnected networks, and can be used to identify potential time windows and/or modes of therapeutic intervention.

To summarize, the focus of our study was to reverse engineer multinodal neural networks with controllable afferent-efferent connectivity. Neural microcircuits in the brain, such as cortical-hippocampal networks, are profoundly complex systems with an intricate microarchitecture involving distinct subpopulations of neurons and other cells. Our approach clearly illustrates the robustness and potential of advanced neuroengineering for recapitulating and longitudinally monitoring complex network dynamics *in vitro* at the micro- and mesoscale. Furthermore, it demonstrates the power of utilizing complex network analysis approaches, such as information- and graph theory, to characterize fundamental network attributes and their importance for information processing. This enables the study of complex network dynamics in healthy and perturbed conditions, including neuropathological processes in adult neural networks. Combination with other cell types of interest thus opens for a wide range of applications in biomedical disease modelling. The potential impact of our advanced reverse engineering approach is thus manifold. It represents an integrated multidisciplinary methodology for advanced disease modelling that can accelerate elucidation of mechanistic causes of disease, pinpoint critical time points for therapeutic intervention, and support testing of the efficacy of such interventions with a view to clinical translation.

## Materials and methods

### Experimental design

**Fig. 7:**
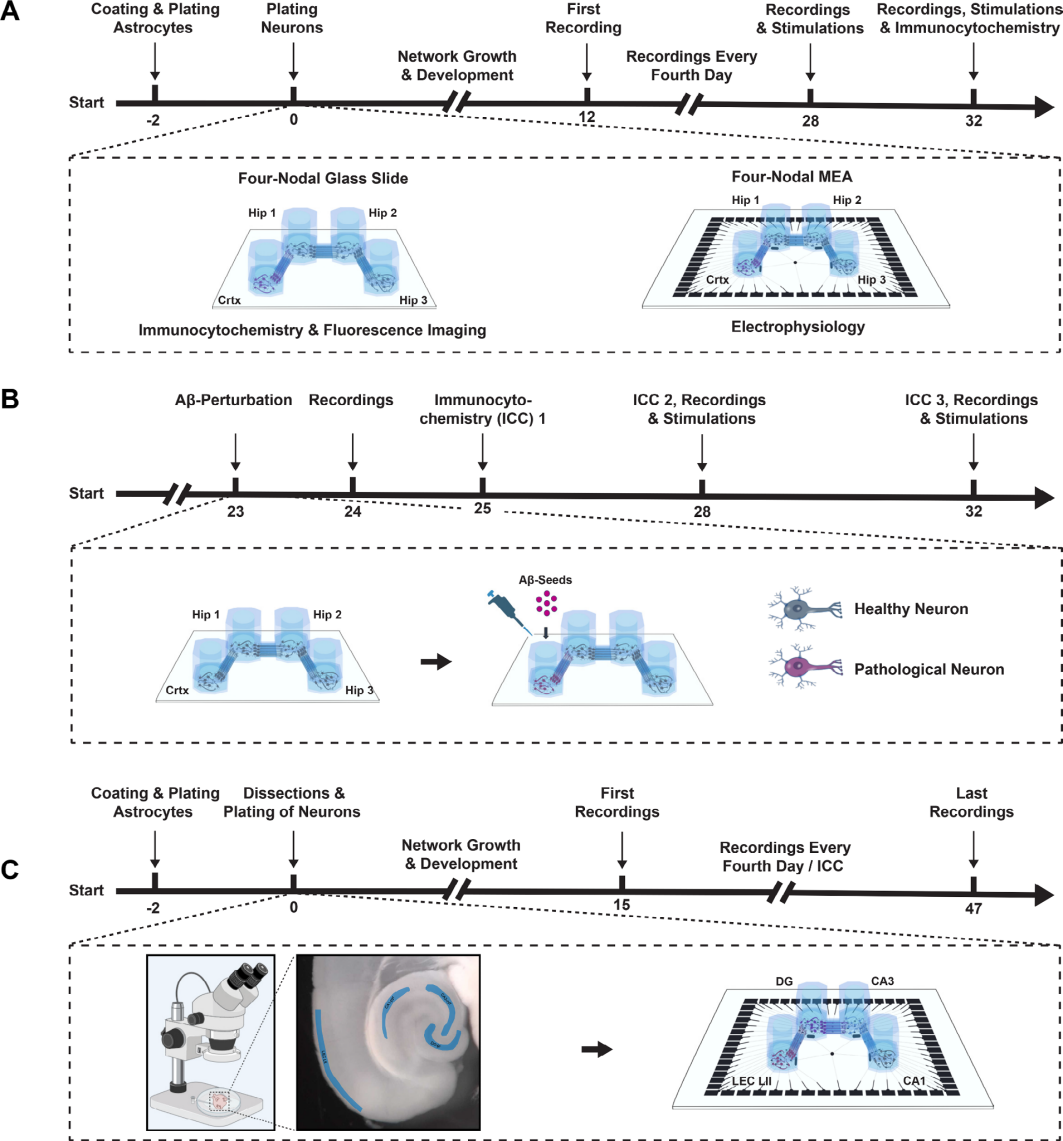
Experimental timelines. (**A**) Four-nodal microfluidic chips were interfaced with either glass coverslips for immunocytochemistry (ICC) or microelectrode arrays (MEAs) for electrophysiology. All interfaces were coated with Poly-L-Ornithine and laminin, and a feeder layer of rat cortical astrocytes were plated two days prior to plating of commercially available rat cortical (node 1) and hippocampal (nodes 2-4) neurons. Spontaneously evoked activity was recorded every fourth day from 12 to 32 DIV, and electrical stimulations were induced after the recordings at 28 and 32 DIV. (**B**) For perturbed networks, all steps were identical up to 23DIV. At this point, amyloid beta (A$\beta$) fragments were added to the cortical node. ICC was conducted 2, 5 and 9 days after perturbation to assess the change in concentration of amyloid beta oligomers in all nodes. Recordings were furthermore conducted 1, 5 and 9 days after the perturbation to evaluate the impact on the network functionality. (**C**) To reconstruct adult entorhinal-hippocampal circuits within the four-nodal microfluidic chips, brains of adult mice and rats were extracted and horizontally sectioned before dissection of the lateral entorhinal cortex layer II, and hippocampal subregional layers (dentate gyrus granular layer, and CA3- and CA1 pyramidal layers). Dissected entorhinal and hippocampal tissues were next dissociated into single cells enzymatically and manually by trituration. LEC LII, DG-gl, CA3-pyr and CA1-pyr were plated in nodes 1-4, respectively. A horizontally cut brain section viewed under a stereoscope using fiber optic illumination for contrast with delineation of entorhinal and hippocampal subregions is illustrated below the timeline. Lateral entorhinal cortex layer II (LEC LII) and hippocampal dentate gyrus granular layer (DG-gl), CA3 pyramidal layer (CA3-pyr) and CA1 pyramidal layer (CA1-pyr) are highlighted in blue.

Experimental timelines including a schematic of the microfluidic MEA design can be seen in Fig. 7. Our neural cultures comprised either commercially available rat embryonic cortical and hippocampal neurons (hereafter referred to as cortical-hippocampal networks), or lateral entorhinal cortex layer II neurons (LEC-LII), Dentate Gyrus granular neurons (DG-gl), CA3 pyramidal neurons (CA3-pyr) and CA1 pyramidal neurons (CA1-pyr), freshly dissected from adult McGill-R-Thy1-APP AD-model rats or APP/PS1 AD-model mice (hereafter referred to as adult entorhinal-hippocampal AD networks). The method for the dissection and culturing of adult hippocampal neurons was refined from our previously reported protocol for adult lateral-most LEC LII (lLEC-LII) neurons from APP/PS1 model mice^43^. For all cultures, coating and establishment of an astrocytic feeder layer was conducted using the same protocol.

### Design & fabrication of microdevices

The four-nodal microdevices were designed using Clewin 4 (WieWeb Software, Enschede). Each node was 5 mm in diameter and ${60}\,{\upmu }{\hbox {m}}$ high. Furthermore, the nodes were connected by 20 microtunnels. To induce unidirectional axonal outgrowth between the nodes, a geometrical design inspired by the Tesla valve was implemented within the microtunnel architecture^24^. All tunnels were ${350}\,{\upmu }{\hbox {m}}$  long, ${10}\,{\upmu }{\hbox {m}}$  wide and ${5}\,{\upmu }{\hbox {m}}$  high. The design also had spine structures on the postsynaptic side to misguide axons trying to enter the microtunnel inlets. 50 nanoporous platinum electrodes of ${50}\,{\upmu }{\hbox {m}}$ diameter were positioned evenly spread across the four nodes. Additionally, 9 electrodes were positioned within the microfluidic tunnels to confirm active connections between the nodes. Individual reference electrodes were embedded in the glass substrate within each of the four chambers on the platforms, with all four reference electrodes wired together and connected to the same contact pad on the MEAs. The design, as well as an image of a fabricated microdevice can be seen in Fig. S5. Fabrication of all microdevices was conducted according to our recently reported protocol^24^.

### Coating of culturing platforms

Prior to coating, all microdevices were sterilized in UV light overnight in a biosafety cabinet. Consecutively, the distilled (DI) water in the devices was replaced by Poly-L-Ornithine solution (PLO) (Sigma-Aldrich, A-004-C) at a concentration of ${0.1}\,{\hbox {mg/mL}}$  and incubated at $37^{\circ }\hbox {C}$, 5 % $\hbox {CO}_2$ for 2 h *or* overnight at ${4}^{\circ }{\hbox {C}}$. Subsequently, all PLO was discarded and the microdevices rinsed three times for 10 min with milli-Q (MQ) water. After the last rinse, laminin solution consisting of ${16}\,{\upmu }{\hbox {mg/mL}}$  natural mouse laminin (Gibco™, 23017015) in phosphate-buffered saline (PBS, Sigma-Aldrich, D8537) was added and the microdevices incubated at ${37}^{\circ }{\hbox {C}}$, 5 % ${\hbox {CO}_2}$ for 2 h. A hydrostatic pressure gradient was established during all coating to ensure flow of the coating solution through the microtunnels. This was achieved by filling the chambers with an unequal amount of coating solution.

### Seeding of astrocyte feeder layer

For the astrocytic feeder layer, a solution consisting of DMEM, low glucose (Gibco™, 11885084) supplemented with 15 % Fetal Bovine Serum (Sigma-Aldrich, F9665) and 2 % Penicillin-Streptomycin (Sigma-Aldrich, P4333) was prepared. Next, the coating solution was replaced by the astrocyte media, and rat cortical astrocytes (Gibco™, N7745100) were seeded at a density of ${100}\,{\hbox {cells/mm}}^{2}$, i.e., 2000 cells per microchamber. The astrocytes were allowed to expand for 48 h, before seeding of either embryonic cortical and hippocampal neurons or adult entorhinal and hippocampal neurons.

### Embryonic neurons: plating and maintenance

Neural media consisting of Neurobasal Plus Medium (Gibco™, A3582801) supplemented with 2 % B27+ (Gibco™, A358201), 1 % GlutaMAX (Gibco™, 35050038) and 2 % Penicillin-Streptomycin (Sigma-Aldrich, P4333) was prepared. Rock Inhibitor (Y-27632 dihydrochloride, Y0503, Sigma-Aldrich) was additionally added to the media during the first two days of neural growth at a concentration of 0.1 % to increase neural survival. Rat cortical neurons from Sprague Dawley rats (Gibco, A36511) were plated at a density of ${1000}\,{\hbox {cells/mm}}^{2}$, equaling 20 000 cells in the first node of each microfluidic interface. The cortical neurons were thereafter allowed to settle in an incubator for 3 h before plating the hippocampal neurons. Next, rat hippocampal neurons from Sprague Dawley rats (Gibco, A36513) were plated at a density of ${1000}\,{\hbox {cells/mm}}^{2}$ in the three remaining nodes of each microfluidic interface. Half the neural media was replaced with fresh neural media 4 h after plating, and again after 24 h. From here on, half the neural media was replaced every second day throughout the experimental period.

### Viral transductions

Viral transductions for inducing ubiquitous expression of fluorescent markers were used to verify unidirectional structural connectivity between the nodes, according to our previously reported method^24^. Viral vectors were prepared in-house at the Viral Vector Core Facility, NTNU. At 11 DIV, neural networks (n = 4) were transduced with AAV 2/1 serotype viruses loaded with either pAAV-CMV-beta Globin intron-EGFP-WPRE-PolyA or pAAV-CMV-beta Globin intron-mCherry-WPRE-PolyA under a CMV promoter to ubiquitously express GFP (nodes 1 and 3) or mCherry (nodes 2 and 4). 3/4 of the media in nodes 1 and 3 were removed to create a hydrostatic pressure gradient across the nodes, and viruses for expression of GFP were added at a concentration of $5e^2$ viruses/cell (i.e. $1e^7$ viruses per node). The cells were subsequently incubated for 3 h at ${37}^{\circ }{\hbox {C}}$ and 5 % $\text {CO}_2$. Next, nodes 1 and 3 were filled all the way up with cell media, and 3/4 of the media in nodes 2 and 4 was removed before adding viruses to express mCherry at a concentration of $5e^2$ viruses/cell. After 3 h of incubation, these nodes were also filled all the way up with media. Imaging of the networks were conducted at 28 DIV.

### Amyloid beta perturbations

At 23 DIV, the cortical node in six four-nodal networks were perturbed using A$\beta$-fragments. Due to the study’s complexity, experimental data were collected in two separate rounds: one for the control networks (n = 6) and another for the perturbed networks (n = 6). For both rounds, the same batch of astrocytes and neurons were utilized, but from different vials provided by the vendor (Gibco). 1 mg Beta-Amyloid Peptide (1-42, human) (ab120301, abcam) was dissolved in ${100}\,{\upmu }{\hbox {L}}$ 1 % ${\hbox {NH}_4{\hbox {OH}}}$ (458680025, Thermo Scientific). The solution was furthermore diluted to 2 mM in neuronal media. This concentration was chosen based on previous literature^79^, and initial concentration testing (Figs. S6 and S7), to induce A$\beta$-oligomerization while still retaining network viability over prolonged periods of time. Subsequently, half the cell media in the cortical node was replaced by 2 mM A$\beta$-solution. Immunocytochemistry was conducted 2, 5 and 9 days after the perturbations to assess the change in concentration of A$\beta$-deposits in all nodes.

### Animals models and genotyping

All animal experiments comply with the ARRIVE guidelines^80^, were approved by the Norwegian Food Safety Authority and carried out in accordance with the EU (European Union) Directive 2010/63/EU for animal experiments. The animals were held in standard lab cages (up to 5 animals per cage), with temperatures of 22 ± ${2}^{\circ }{\hbox {C}}$, and kept at a light/dark cycle of 12:12 hours with access to food and water *ad libitum*. For optimization of the protocol we used a total of 43 adult McGill-R-Thy1-APP rats, either homozygote (+/+), heterozygote (+/-) or negative genotype (-/-), including 22 female and 21 male. Of these, we collected data from 14 rats (8 female and 6 male) and from a total of 2 female, adult APP/PS1 mice. See Supplementary Table S2 for a total overview of sex, age and genotype of all animals.

The APP/PS1 mouse model is a transgenic familial AD-model, with a C57BL/6J genetic background, co-expressing mutated human amyloid precursor protein (hAPP) (KM670/671NL) and mutated human presenilin 1 (L166). Both mutated proteins are driven by expression cassettes under the Thy1 promoter. This causes increased levels of A$\beta$ leading to formation of extracellular cortical A$\beta$-deposits starting already from around 6 weeks of age^81^. The APP/PS1 mice were genotyped using a KAPA-kit as described in Hanssen *et al.*^43^. The McGill-R-Thy1-APP rat model is a transgenic familial AD model with a Wistar (HsdBrl:WH) genetic background, co-expressing mutated hAPP (APP K670M671delinsNL) and (APP V717F)^82^. The McGill-R-Thy1-APP rats were genotyped using quantitative PCR (qPCR) and genomic DNA isolated from ear tissue as described in previous studies^83^. Both homozygous and heterozygous rats were included in the study, in addition to controls.

### Adult neurons: dissection

All tools were sterilized by autoclaving before use. Both mice and rats were deeply anesthetized using 5 % isoflurane gas (Abbott Lab, 05260-05) and checked for absence of reflexes before the head was decapitated with a guillotine for rats or surgical scissors (FST, 14007-14) for mice. Subsequently, dissection and sectioning of the brain were conducted as described previously for both mice and rats. See Hanssen *et al.* for a detailed description of the procedure^43^. Brains were extracted in a petri dish filled with Hanks Balanced Salt Solution (Thermo Fischer Scientific, 88284) kept on ice. Extracted brains were sectioned horizontally on a Leica VT1000 S vibratome (Leica Biosystems) (thickness of ${300}\,{\upmu }{\hbox {m}}$, frequency of 5.3 Hz and speed of 8 mm/s) with 0.2 % AGAR (VWR, 20767.298) supporting the caudal end of the brain. Sectioned brain slices were immediately transferred to wells of a sterile 24-well plate held on ice. Sections were further transferred to a petri dish held on ice and viewed under a stereoscope with fiber optic lamps, allowing for sufficient contrast of the cytoarchitectonic landmarks. Dissection of the lateral entorhinal cortex layer II (LEC-LII) was conducted as described in our recent study for both mice and rats^43^. Additionally, the dentate gyrus granular layer (DG-gl), CA3 pyramidal (pyr) and CA1-pyr layers were dissected (Fig. 7C). The DG-gl can be separated from the molecular layer and hilus using its densely packed layer of granular neurons. Similarly, the pyramidal layer in CA3 and CA1 consists of densely packed pyramidal neurons that can be distinguished from superficial layers lucidum, radiatum and lacunosum-moleculare and deep layer oriens^33^. All dissected strips of tissue were placed by region in four separate sterile 15 mL tubes filled with dissection media (HABG-P) consisting of Hibernate-A (Thermo Fischer Scientific, A1247501) supplemented with 2 % B27+ (Gibco™, A3582801), 2.5 % GlutaMAX (Gibco™, 35050061) and 1 % Penicillin-streptomycin (Sigma-Aldrich, P4333), placed on ice.

### Adult neurons: plating and maintenance

All dissected tissue was rinsed for debris three times by adding and removing 3 mL of HABG-P. Following this, all media was discarded and the tissue was dissociated for 15 min at ${37}^{\circ }{\hbox {C}}$, 5 % ${\hbox {CO}_2}$, 20 % ${\hbox {O}_2}$ in Neural Isolation Enzyme Papain (Thermo Fischer Scientific, 88285) reconstituted in Hank’s Balanced Salt solution (Thermo Fischer Scientific, 88284) as described by the manufacturer (Thermo Fischer Scientific, MAN0011896). Subsequently, all dissociation enzyme was discarded, ${100}\,{\upmu }{\hbox {L}}$ HABG-P was added and the tissue was manually triturated 10 times by using a ${100}\,{\upmu }{\hbox {L}}$ pipette tip. Further, HABG-P was topped up to a total of ${3}\,{\upmu }{\hbox {L}}$ and the vials of tissue were centrifuged at x200 g for 2 min in room temperature. After centrifugation, HABG-P was discarded and ${100}\,{\upmu }{\hbox {L}}$ neural media was added before manual trituration 10 times using a ${100}\,{\upmu }{\hbox {L}}$ pipette tip. The neural media consisted of Neurobasal Plus Medium (Gibco™, A3582801) supplemented with 2 % B27+ (Gibco™, A358201), 1 % GlutaMAX (Gibco™, 35050038), 2 % Penicillin-Streptomycin (Sigma-Aldrich, P4333), 0.1 % Rock Inhibitor (Y-27632 dihydrochloride, Y0503, Sigma-Aldrich) and 10 % Fetal Bovine Serum (Sigma Aldrich, 12106C). For adult neurons derived from mice, 0.1 % BDNF (Neurotrophins, 450-02) was added to the neural media. For adult neurons derived from rats, 0.1 % FGF2 (Thermo Fisher Scientific, 13256-029) was added to the neural media. Neurons were plated at a density of ${750}\,{\hbox {cells/mm}}^{2}$, equaling 15 000 cells in each node of the microfluidic chip. Half the neural media was replaced every second day throughout the experimental period.

### Immunocytochemistry

For immunocytochemistry, both cortical-hippocampal and adult entorhinal-hippocampal neurons were plated in microfluidic platforms bonded to glass coverslips (VWR International, 24x24 mm No. 1 Menzel-Gläser). Fixation of cortical-hippocampal cultures was conducted using glyoxal solution based on the protocol by Richter *et al.*^84^. This fixative consisted of 20 % ethanol absolute (Kemetyl, 100 %), 8.0 % Glyoxal solution (Sigma-Aldrich, 128465) and 1 % acetic acid (Sigma-Aldrich, 1.00063) in MQ-water. Fixation of adult entorhinal-hippocampal cultures was conducted using PBS (Sigma-Aldrich, D8537) containing 0.4 M phosphate buffer and 4 % freshly depolymerized paraformaldehyde (PFA; Sigma-Aldrich, P6148). The cultures were washed with PBS to remove debris, before the fixative was applied for 15 min at room temperature. Subsequently, all chambers were washed with PBS 3 times for 15 min each. Next, 0.5 % Triton-X (Sigma-Aldrich, 1086431000) diluted in PBS was applied to permeabilize the cells. All chambers were again washed twice with PBS before a blocking solution consisting of 5 % goat serum (Abcam, ab7481) diluted in PBS was added to the cultures, and the cultures incubated at room temperature on a shaking table at 30 rpm for 1 h. Primary antibody solutions were prepared in PBS with 5 % goat serum, and antibody concentrations according to the ones listed in Table 1. Cultures were placed on a shaker table at 30 rpm at ${4}^{\circ }{\hbox {C}}$ overnight. Following this, the primary antibody solution was removed, and the cell cultures were rinsed three times with PBS for 15 min each. Next, a secondary antibody solution consisting of 0.2 % secondaries and 5 % goat serum diluted in PBS was added to the cultures. The cultures were left to rest on a shaker table at 30 rpm for 3 h at room temperature. Prior to applying the secondary antibody solution, the prepared solutions were centrifuged at 6000 rpm for at least 15 min to remove precipitates. Subsequently, the secondary antibody solution was replaced by Hoechst (Abcam, ab228550) at a concentration of 0.1 % diluted in PBS, and the cultures left on a shaker table for another 30 min. Eventually, all cultures were washed three more times in PBS, then twice in MQ water prior to imaging.

**Table 1:** Antibodies and concentrations used for Immunocytochemistry.

Marker	Catalogue number	Concentration
$\beta$3-Tubulin	Ab78078	1/1000
CAMKIIa	MA3-918 (Invitrogen)	1/250
GAD 65/67	Ab183999	1/100
GAP-43	9264 (Sigma-Aldrich)	1/500
GFAP	Ab7260	1/1000
MAP2	Ab5392	1/5000
McSA1	MM-0015-P (MédiMabs)	1/750
NeuN	Ab279295	1/500
Neurofilament Heavy	Ab4680	1/5000
PSD95	Ab13552	1/250
Synaptophysin	Ab32127	1/500
Reelin	Ab78540	1/10000

All antibodies were purchased from Abcam unless otherwise stated.

All images were acquired using an EVOS M5000 microscope (Invitrogen). The microscope was equipped with DAPI (AMEP4650), CY5 (AMEP4656), GFP (AMEP4651) and TxRed (AMEP4655) LED light cubes and Olympus UPLSAP0 4X/0.16 NA and 20x/0.75 NA objectives. Post-processing of images was conducted in ImageJ/Fiji or Adobe Photoshop 2020.

### Quantification of amyloid beta deposits

For quantification of amyloid beta deposits, ImageJ/Fiji was utilized. For each Day Post Perturbation (DPP; 2, 5, and 9), a total of four images were used for quantification in both perturbed and control networks, except for DPP 2 and 5 for control networks, where only two images were used. Each image was captured with a 20x objective, covering an area of 2046 pixels, equivalent to ${2803}\,{\upmu }{\hbox {m}}$. All images were converted to 8-bit and thresholded using maximum entropy. Furthermore, the images were binarized, and particles separated using the watershed function. The built-in particle analysis function was subsequently used to count the number and size of the A$\beta$-seeds, and only particles above ${5}\,{\upmu }{\hbox {m}}$ were included in the final analysis. The threshold was set this high to assure only aggregates of amyloid beta were included in the analysis, and not noise caused by antibody precipitates or autofluorescence. This provided the best comparison of amyloid beta levels between networks stained at different time points.

### Imaging and quantification of neurite length

For quantification of neurite length, phase contrast images acquired using a Zeiss Axio Vert V.1 brightfield 20x/53 WD/0.4 NA objective with an Axiocam 202 mono were used. For analysis, Neurolucida (MFB Bioscience) was used to quantify neurite length of the adult neurons.

### Electrophysiological recordings

A MEA2100 workstation (Multichannel Systems) was utilized for all recordings with a sampling rate of 25000 Hz. An external temperature controller (TC01, Multichannel Systems) was used to maintain the temperature at ${37}^{\circ }{\hbox {C}}$. All cultures were allowed to equilibrate at the workstation for 5 min prior to recordings, and recordings lasted for 15 min for cortical-hippocampal networks and 10 min for adult entorhinal-hippocampal neural networks. A 3D-printed plastic cover with a gas-permeable membrane was used to keep the neural networks in a sterile environment during the recordings.

### Electrical stimulations

Stimulations of cortical-hippocampal neurons were performed following the recordings at 28 and 32 DIV using a train of 60 spikes at ± 800 mV (positive phase first) of ${200}\,{\upmu }{\hbox {s}}$ duration with an interspike interval of 5 s. The most active electrode measured in spikes per second in the cortical cell population was chosen for stimulations. This was done to assure that the electrode chosen for stimulation had sufficient coupling with a neuron to induce activity.

### Data analysis

All data preprocessing and analysis was conducted in Matlab R2021b, and graphs were plotted using the matlab function linspecer^85^, based on the color palette colorBrewer^86^. A 4th order Butterworth bandpass filter was used to remove noise below 300 Hz and above 3000 Hz, and a notch filter to remove noise at 50 Hz from the power supply mains. All filters were run using zero-phase digital filtering. For spike detection, the Precise Timing Spike Detection (PTSD) algorithm developed by Maccione *et al.*^87^ was utilized. A threshold of 9 times the standard deviation was chosen for the cortical-hippocampal cultures, with a maximum peak duration of 1 ms and a refractory period of 1.6 ms. For the adult entorhinal-hippocampal cultures, a standard deviation of 7.5 was chosen due to the lower signal-to-noise ratio of the activity in these cultures overall. The *SpikeRasterPlot* function developed by Kraus^88^ was adapted and used for creating raster plots.

Burst detection was conducted using the logISI algorithm developed by Pasquale *et al.*^44^. A minimum of four consecutive spikes was set as a hard threshold for a burst to be detected, and the maximum interspike interval to 100 ms. Network bursts were detected using the logIBEI approach^89^, with a minimum of 10 % of all active electrodes required to exhibit bursting activity for a network burst to be classified. To assess whether activity originating in the cortical node propagated to the hippocampal nodes, the sequence of electrode activation during network bursts was analyzed. For a network burst to be classified as having propagated through all four nodes, activity had to be detected sequentially in a feedforward direction, starting from the cortical node and progressing through hippocampal nodes 1, 2, and 3. Coherence index, a metric for assessing network synchrony, was calculated as the ratio of the standard deviation to the mean of the instantaneous firing rate^90^.

After binning the data into 50 ms time bins, functional connectivity was analyzed using cross-correlation. For each calculation, the maximum cross-correlation coefficient was computed between the binned spike trains of each node and up to 500 ms time-shifted copies of the activity from other nodes, with a maximum lag of 10 bins (500 ms). The cross-correlation was normalized so that the autocorrelations at zero lag were equal to 1, using the *coeff* specification in the Matlab function *xcorr*. Cross-correlation was selected as the correlation metric to capture the correlation between nodes that are farther apart, where the propagation of activity might take longer (e.g., between the cortical node and hippocampal node 3). The cross-correlation within nodes was evaluated by calculating the correlation between two and two electrodes within an individual node, and then assessing the average value of these correlations to get a single correlation value for each node. For the correlation between nodes, the spike trains from all electrodes in each node were combined, before the correlation between the combined spike trains between nodes was evaluated. This approach was adopted to minimize variability between nodes caused by differences in the number of electrodes detecting activity, given the limited number of electrodes per node. Since extracellular electrodes predominantly capture signals from the perisomatic region of neurons (within 50-${100}\,{\upmu }{\hbox {m}}$ of the soma^9^), a single neuron is typically detected by only one electrode on these platforms. Furthermore, the 50 ms bin size used in the analysis ensures that activity from multiple neurons near an electrode is averaged within the same time bin during network bursts. This averaging reduces the potential impact of multiunit activity on internodal correlations and, consequently, diminishes the necessity for spike sorting in this context.

To visualize key network features, graphs were plotted using the electrodes as nodes, and cross-correlation as the edges (i.e. lines connecting the individual nodes). Edges with weaker connectivity than 0.1 were removed from the graph representations to highlight only the strongest, direct connections. Firing rate (Hz) was furthermore used to represent the node color and pagerank centrality the node size. The Louvain algorithm was used to perform community detection, delineating the nodes into distinct communities based on which nodes were more highly interconnected with each other than the rest of the network^91^. This analysis was used to determine the maximized modularity value of the networks. A high modularity value indicates that the network has dense internal connections within nodes and sparse connections between different nodes. The node edge color of the graphs were furthermore used to showcase which nodes belonged to the same community. The modularity value was used to assess how easily the algorithm managed to classify the nodes into distinct communities. All graph theoretical measures were calculated using the Brain Connectivity Toolbox developed by Rubinov & Sporns^92^.

To remove stimulation artifacts, stimulation data was run through the SALPA filter developed by Wagenaar *et al.*^93^. Additionally, 15 ms of the filtered data was blanked following each stimulation time point. Subsequently, the data was binned into 20 ms time bins, and peristimulus time histograms of the average response of the stimulations in each node plotted.

### Statistical analysis

Generalized Linear Mixed-Effect Models (GLMMs) were used to assess differences in network parameters between control networks and networks perturbed with A$\beta$-fragments. The GLMMs were generated using SPSS version 29.0.0.0. The network type, i.e. perturbed networks from DIV 24 to 32 (i.e. after perturbation) or control networks (i.e. all other data points for both perturbed and unperturbed networks) were used as a fixed effect. Considering that both control and perturbed networks received identical treatment until the point of perturbation, it was considered more appropriate to compare post-perturbation data points from the perturbed networks (represented by the pink bar in the GLMM) to the data points from the control networks throughout the experimental period and the perturbed networks before perturbation (represented by the blue bar in the GLMM). The network age was furthermore used as a random effect, and the network feature of interest as a target. To fit the data, a gamma distribution with a log link function was chosen based on the Akaike information criterion. Sequential Bonferroni adjustment was selected for multiple comparison. All other statistical analyses were conducted using a two-sided Wilcoxon rank sum test with the Matlab function *ranksum*.

## Supplementary Information

Supplementary Information.

## Data Availability

The data supporting the findings of this study are available from the corresponding author upon reasonable request.

## References

[1] Wagenaar, D. A., Pine, J. & Potter, S. M. An extremely rich repertoire of bursting patterns during the development of cortical cultures. *BMC Neurosci.* **7**, 11. 10.1186/1471-2202-7-11 (2006). 16464257 10.1186/1471-2202-7-11 PMC1420316

[2] Chiappalone, M., Bove, M., Vato, A., Tedesco, M. & Martinoia, S. Dissociated cortical networks show spontaneously correlated activity patterns during in vitro development. *Brain Res.* **1093**(1), 41–53. 10.1016/j.brainres.2006.03.049 (2006). 16712817 10.1016/j.brainres.2006.03.049

[3] Heiney, K. et al. Neuronal avalanche dynamics and functional connectivity elucidate information propagation in vitro. *Front. Neural Circuits* **16**, 980631. 10.3389/fncir.2022.980631 (2022). 36188125 10.3389/fncir.2022.980631 PMC9520060

[4] Poli, D., Wheeler, B. C., DeMarse, T. B. & Brewer, G. J. Pattern separation and completion of distinct axonal inputs transmitted via micro-tunnels between co-cultured hippocampal dentate, ca3, ca1 and entorhinal cortex networks. *J. Neural Eng.* **15**(4), 046009. 10.1088/1741-2552/aabc20 (2018). 29623900 10.1088/1741-2552/aabc20 PMC6021217

[5] Taylor, A. M. et al. Microfluidic multicompartment device for neuroscience research. *Langmuir* **19**(5), 1551–56. 10.1021/la026417v (2003). 20725530 10.1021/la026417v PMC2923462

[6] Taylor, A. M. et al. A microfluidic culture platform for cns axonal injury, regeneration and transport. *Nat. Methods* **2**, 599–605. 10.1038/nmeth777 (2005). 16094385 10.1038/nmeth777 PMC1558906

[7] Pan, L. et al. An in vitro method to manipulate the direction and functional strength between neural populations. *Front. Neural Circuits*[SPACE]10.3389/fncir.2015.00032 (2015). 26236198 10.3389/fncir.2015.00032 PMC4500931

[8] Brofiga, M., Pisano, M., Tedesco, M., Boccaccio, A. & Massobrio, P. Functional inhibitory connections modulate the electrophysiological activity patterns of cortical-hippocampal ensemble. *Cereb. Cortex* **32**(9), 1866–1881. 10.1093/cercor/bhab318 (2022). 34535794 10.1093/cercor/bhab318

[9] Obien, M. E. J., Deligkaris, K., Bullmann, T., Bakkum, D. J. & Frey, U. Revealing neuronal function through microelectrode array recordings. *Front Neurosci.*[SPACE]10.3389/fnins.2014.00423 (2015). 25610364 10.3389/fnins.2014.00423 PMC4285113

[10] Mossink, B. et al. Human neuronal networks on micro-electrode arrays are a highly robust tool to study disease-specific genotype-phenotype correlations in vitro. *Stem Cell Rep.* **16**(9), 2182–2196. 10.1016/j.stemcr.2021.07.001 (2021). 10.1016/j.stemcr.2021.07.001 PMC8452490 34329594

[11] Keller, J. M. & Frega, M. Past, present, and future of neuronal models in vitro. *Adv Neurobiol.* **22**, 3–17. 10.1007/978-3-030-11135-9_1 (2019). 31073930 10.1007/978-3-030-11135-9_1

[12] DeMarse, T. B., Pan, L., Alagapan, S., Brewer, G. J. & Wheeler, B. C. Feed-forward propagation of temporal and rate information between cortical populations during coherent activation in engineered in vitro networks. *Front. Neural Circuits*[SPACE]10.3389/fncir.2016.00032 (2016). 27147977 10.3389/fncir.2016.00032 PMC4840215

[13] Benito, N. et al. Spatial modules of coherent activity in pathway-specific lfps in the hippocampus reflect topology and different modes of presynaptic synchronization. *Cereb. Cortex* **24**(7), 1738–52. 10.1093/cercor/bht022 (2014). 23395845 10.1093/cercor/bht022

[14] Withers, G. S., James, C. D., Kingman, C. E., Craighead, H. G. & Banker, G. A. Effects of substrate geometry on growth cone behavior and axon branching. *J. Neurobiol.* **66**(11), 1183–94. 10.1002/neu.20298 (2006). 16858695 10.1002/neu.20298

[15] Dent, E. W., Gupton, S. L. & Gertler, F. B. The growth cone cytoskeleton in axon outgrowth and guidance. *Cold Spring Harb. Perspect. Biol.* **3**(3), a001800. 10.1101/cshperspect.a001800 (2011). 21106647 10.1101/cshperspect.a001800 PMC3039926

[16] Gangatharan, G., Schneider-Maunoury, S. & Breau, M. A. Role of mechanical cues in shaping neuronal morphology and connectivity. *Biol. Cell* **110**(6), 125–36. 10.1111/boc.201800003 (2018). 29698566 10.1111/boc.201800003

[17] Peyrin, J. M. et al. Axon diodes for the reconstruction of oriented neuronal networks in microfluidic chambers. *Lab Chip* **11**(21), 3663–73. 10.1039/c1lc20014c (2011). 21922081 10.1039/c1lc20014c

[18] Malishev, E. et al. Microfluidic device for unidirectional axon growth. *J. Phys. Conf. Ser.* **643**, 012025. 10.1088/1742-6596/643/1/012025 (2015).

[19] le Feber, J., Postma, W., de Weerd, E., Weusthof, M. & Rutten, W. L. Barbed channels enhance unidirectional connectivity between neuronal networks cultured on multi electrode arrays. *Front. Neurosci.* **9**, 412. 10.3389/fnins.2015.00412 (2015). 26578869 10.3389/fnins.2015.00412 PMC4630305

[20] Gladkov, A. et al. Design of cultured neuron networks in vitro with predefined connectivity using asymmetric microfluidic channels. *Sci. Rep.* **7**(1), 15625. 10.1038/s41598-017-15506-2 (2017). 29142321 10.1038/s41598-017-15506-2 PMC5688062

[21] Na, S. et al. Microfluidic neural axon diode. *Technology* **4**(4), 240–8. 10.1142/S2339547816500102 (2016).

[22] Holloway, P. M. et al. Asymmetric confinement for defining outgrowth directionality. *Lab Chip* **19**(8), 1484–89. 10.1039/c9lc00078j (2019). 30899932 10.1039/c9lc00078j

[23] Renault, R., Durand, J.-B., Viovy, J.-L. & Villard, C. Asymmetric axonal edge guidance: a new paradigm for building oriented neuronal networks. *Lab Chip* **16**(12), 2188–91. 10.1039/c6lc00479b (2016). 27225661 10.1039/c6lc00479b

[24] Winter-Hjelm, N., Tomren, Å. B., Sikorski, P., Sandvig, A. & Sandvig, I. Structure-function dynamics of engineered, modular neuronal networks with controllable afferent-efferent connectivity. *J. Neural Eng.* **20**, 046024. 10.1088/1741-2552/ace37f (2023). 10.1088/1741-2552/ace37f 37399808

[25] Vakilna, Y. S., Tang, W. C., Wheeler, B. C. & Brewer, G. J. The flow of axonal information among hippocampal subregions: 1. Feed-forward and feedback network spatial dynamics underpinning emergent information processing. *Front. Neural Circuits* **15**, 660837. 10.3389/fncir.2021.660837 (2021). 34512275 10.3389/fncir.2021.660837 PMC8430040

[26] Yamamoto, H. et al. Impact of modular organization on dynamical richness in cortical networks. *Sci. Adv.* **4**, 11. 10.1126/sciadv.aau4914 (2018). 10.1126/sciadv.aau4914 PMC6235526 30443598

[27] Park, M. U. et al. Collective dynamics of neuronal activities in various modular networks. *Lab Chip* **21**(5), 951–61. 10.1039/d0lc01106a (2021). 33475100 10.1039/d0lc01106a

[28] van de Wijdeven, R. et al. Structuring a multi-nodal neural network in vitro within a novel design microfluidic chip. *Biomed. Microdevices* **20**, 9. 10.1007/s10544-017-0254-4 (2018). 29294210 10.1007/s10544-017-0254-4

[29] Dworak, B. J. & Wheeler, B. C. Novel mea platform with pdms microtunnels enables the detection of action potential propagation from isolated axons in culture. *Lab Chip* **9**(3), 404–10. 10.1039/b806689b (2009). 19156289 10.1039/b806689b PMC2790813

[30] Levy, O., Ziv, N. E. & Marom, S. Enhancement of neural representation capacity by modular architecture in networks of cortical neurons. *Eur. J. Neurosci.* **35**(11), 1753–60. 10.1111/j.1460-9568.2012.08094.x (2012). 22507055 10.1111/j.1460-9568.2012.08094.x

[31] Baruchi, I., Volman, V., Raichman, N., Shein, M. & Ben-Jacob, E. The emergence and properties of mutual synchronization in in vitro coupled cortical networks. *Eur. J. Neurosci.* **28**(9), 1825–35. 10.1111/j.1460-9568.2008.06487.x (2008). 18973597 10.1111/j.1460-9568.2008.06487.x

[32] Shein-Idelson, M., Cohen, G., Ben-Jacob, E. & Hanein, Y. Modularity induced gating and delays in neuronal networks. *PLoS Comput. Biol.* **12**(4), e1004883. 10.1371/journal.pcbi.1004883 (2016). 27104350 10.1371/journal.pcbi.1004883 PMC4841573

[33] van Strien, N. M., Cappaert, N. L. & Witter, M. P. The anatomy of memory: An interactive overview of the parahippocampal-hippocampal network. *Nat. Rev. Neurosci.* **10**(4), 272–82. 10.1038/nrn2614 (2009). 19300446 10.1038/nrn2614

[34] Braak, H. & Braak, E. Neuropathological stageing of Alzheimer-related changes. *Acta Neuropathol.* **82**(4), 239–59. 10.1007/bf00308809 (1991). 1759558 10.1007/BF00308809

[35] Thal, D. R., Rüb, U., Orantes, M. & Braak, H. Phases of a beta-deposition in the human brain and its relevance for the development of ad. *Neurology* **58**(12), 1791–800. 10.1212/wnl.58.12.1791 (2002). 12084879 10.1212/wnl.58.12.1791

[36] Vossel, K. A. et al. Seizures and epileptiform activity in the early stages of Alzheimer disease. *JAMA Neurol.* **70**(9), 1158–66. 10.1001/jamaneurol.2013.136 (2013). 23835471 10.1001/jamaneurol.2013.136 PMC4013391

[37] Gómez-Isla, T. et al. Profound loss of layer ii entorhinal cortex neurons occurs in very mild Alzheimer’s disease. *J. Neurosci.* **16**(14), 4491–500. 10.1523/JNEUROSCI.16-14-04491.1996 (1996). 8699259 10.1523/JNEUROSCI.16-14-04491.1996 PMC6578866

[38] Kordower, J. H. et al. Loss and atrophy of layer ii entorhinal cortex neurons in elderly people with mild cognitive impairment. *Ann. Neurol.* **49**(2), 202–13 (2001). 11220740

[39] Scheff, S. W., Price, D. A., Schmitt, F. A. & Mufson, E. J. Hippocampal synaptic loss in early Alzheimer’s disease and mild cognitive impairment. *Neurobiol. Aging* **27**(10), 1372–84. 10.1016/j.neurobiolaging.2005.09.012 (2006). 16289476 10.1016/j.neurobiolaging.2005.09.012

[40] Scheff, S. W., Price, D. A., Schmitt, F. A., DeKosky, S. T. & Mufson, E. J. Synaptic alterations in ca1 in mild Alzheimer disease and mild cognitive impairment. *Neurology* **68**(18), 1501–8. 10.1212/01.wnl.0000260698.46517.8f (2007). 17470753 10.1212/01.wnl.0000260698.46517.8f

[41] Dong, Y., Sameni, S., Digman, M. A. & Brewer, G. J. Reversibility of age-related oxidized free nadh redox states in alzheimer’s disease neurons by imposed external cys/cyss redox shifts. *Sci. Rep.* **9**(1), 11274. 10.1038/s41598-019-47582-x (2019). 31375701 10.1038/s41598-019-47582-x PMC6677822

[42] Dong, Y., Digman, M. A. & Brewer, G. J. Age- and ad-related redox state of nadh in subcellular compartments by fluorescence lifetime imaging microscopy. *Geroscience* **41**(1), 51–67. 10.1007/s11357-019-00052-8 (2019). 30729413 10.1007/s11357-019-00052-8 PMC6423217

[43] Hanssen, K. S., Witter, M. P., Sandvig, A. I. & Kobro-Flatmoen, A. Dissection and culturing of adult lateral entorhinal cortex layer ii neurons from app/ps1 alzheimer model mice. *J. Neurosci. Methods* **390**, 109840. 10.1016/j.jneumeth.2023.109840 (2023). 36948358 10.1016/j.jneumeth.2023.109840

[44] Pasquale, V., Martinoia, S. & Chiappalone, M. A self-adapting approach for the detection of bursts and network bursts in neuronal cultures. *J. Comput. Neurosci.* **29**(1–2), 213–29. 10.1007/s10827-009-0175-1 (2010). 19669401 10.1007/s10827-009-0175-1

[45] Weir, J. S., Christiansen, N., Sandvig, A. & Sandvig, I. Selective inhibition of excitatory synaptic transmission alters the emergent bursting dynamics of in vitro neural networks. *Front. Neural Circuits* **17**, 1020487. 10.3389/fncir.2023.1020487 (2023). 36874945 10.3389/fncir.2023.1020487 PMC9978115

[46] Nunez, J. Differential expression of microtubule components during brain development. *Dev. Neurosci.* **8**(3), 125–41. 10.1159/000112248 (1986). 3533503 10.1159/000112248

[47] Johnson, G. V. W. & Jope, R. S. The role of microtubule-associated protein 2 (map-2) in neuronal growth, plasticity, and degeneration. *J. Neurosci. Res.* **33**(4), 505–12. 10.1002/jnr.490330402 (1992). 1484385 10.1002/jnr.490330402

[48] Tischfield, M. A. et al. Human tubb3 mutations perturb microtubule dynamics, kinesin interactions, and axon guidance. *Cell* **140**(1), 74–87. 10.1016/j.cell.2009.12.011 (2010). 20074521 10.1016/j.cell.2009.12.011 PMC3164117

[49] Oestreicher, A. B., De Graan, P. N. E., Gispen, W. H., Verhaagen, J. & Schrama, L. H. B-50, the growth associated protein-43: modulation of cell morphology and communication in the nervous system. *Prog. Neurobiol.* **53**(6), 627–86. 10.1016/S0301-0082(97)00043-9 (1997). 9447616 10.1016/s0301-0082(97)00043-9

[50] Mullen, R. J., Buck, C. R. & Smith, A. M. Neun, a neuronal specific nuclear protein in vertebrates. *Development* **116**(1), 201–11. 10.1242/dev.116.1.201 (1992). 1483388 10.1242/dev.116.1.201

[51] Schlaepfer, W. W. & Bruce, J. Simultaneous up-regulation of neurofilament proteins during the postnatal development of the rat nervous system. *J. Neurosci. Res.* **25**(1), 39–49. 10.1002/jnr.490250106 (1990). 2108255 10.1002/jnr.490250106

[52] Eng, L. F., Ghirnikar, R. S. & Lee, Y. L. Glial fibrillary acidic protein: Gfap-thirty-one years (1969–2000). *Neurochem. Res.* **25**(9–10), 1439–51. 10.1023/A:1007677003387 (2000). 11059815 10.1023/a:1007677003387

[53] Wiedenmann, B. & Franke, W. W. Identification and localization of synaptophysin, an integral membrane glycoprotein of mr 38,000 characteristic of presynaptic vesicles. *Cell* **41**(3), 1017–28. 10.1016/S0092-8674(85)80082-9 (1985). 3924408 10.1016/s0092-8674(85)80082-9

[54] Cho, K.-O., Hunt, C. A. & Kennedy, M. B. The rat brain postsynaptic density fraction contains a homolog of the drosophila discs-large tumor suppressor protein. *Neuron* **9**(5), 929–42. 10.1016/0896-6273(92)90245-9 (1992). 1419001 10.1016/0896-6273(92)90245-9

[55] Liu, X. B. & Jones, E. G. Localization of alpha type ii calcium calmodulin-dependent protein kinase at glutamatergic but not gamma-aminobutyric acid (gabaergic) synapses in thalamus and cerebral cortex. *PNAS* **93**(14), 7332–6. 10.1073/pnas.93.14.7332 (1996). 8692993 10.1073/pnas.93.14.7332 PMC38984

[56] Erlander, M. G., Tillakaratne, N. J. K., Feldblum, S., Patel, N. & Tobin, A. J. Two genes encode distinct glutamate decarboxylases. *Neuron* **7**(1), 91–100. 10.1016/0896-6273(91)90077-D (1991). 2069816 10.1016/0896-6273(91)90077-d

[57] Feldblum, S., Erlander, M. G. & Tobin, A. J. Different distributions of gad65 and gad67 mrnas suggest that the two glutamate decarboxylases play distinctive functional roles. *J. Neurosci. Res.* **34**(6), 689–706. 10.1002/jnr.490340612 (1993). 8315667 10.1002/jnr.490340612

[58] Luján, R., Shigemoto, R. & López-Bendito, G. Glutamate and gaba receptor signalling in the developing brain. *Neuroscience* **130**(3), 567–80. 10.1016/j.neuroscience.2004.09.042 (2005). 15590141 10.1016/j.neuroscience.2004.09.042

[59] Luhmann, H. J., Fukuda, A. & Kilb, W. Control of cortical neuronal migration by glutamate and gaba. *Front. Cell. Neurosci.*[SPACE]10.3389/fncel.2015.00004 (2015). 25688185 10.3389/fncel.2015.00004 PMC4311642

[60] Behuet, S. et al. Developmental changes of glutamate and gaba receptor densities in wistar rats. *Front. neuroanat.* **13**, 100. 10.3389/fnana.2019.00100 (2019). 31920569 10.3389/fnana.2019.00100 PMC6933313

[61] Martínez–Cerdeño, V., Galazo, M. . J. & Clascá, F. Reelin-immunoreactive neurons, axons, and neuropil in the adult ferret brain: Evidence for axonal secretion of reelin in long axonal pathways. *J. Comp. Neurol.* **463**(1), 92–116. 10.1002/cne.10748 (2003). 12811805 10.1002/cne.10748

[62] Shanahan, M. Dynamical complexity in small-world networks of spiking neurons. *Phys. Rev. E Stat. Nonlin. Soft Matter Phys.*[SPACE]10.1103/PhysRevE.78.041924 (2008). 18999472 10.1103/PhysRevE.78.041924

[63] Rubinov, M., Sporns, O., Thivierge, J.-P. & Breakspear, M. Neurobiologically realistic determinants of self-organized criticality in networks of spiking neurons. *PLoS Comput. Biol.* **7**(6), e1002038. 10.1371/journal.pcbi.1002038 (2011). 21673863 10.1371/journal.pcbi.1002038 PMC3107249

[64] Meunier, D., Lambiotte, R. & Bullmore, E. T. Modular and hierarchically modular organization of brain networks. *Front. Neurosci.* **4**, 200. 10.3389/fnins.2010.00200 (2010). 21151783 10.3389/fnins.2010.00200 PMC3000003

[65] Busche, M. A. et al. Critical role of soluble amyloid-Î2 for early hippocampal hyperactivity in a mouse model of Alzheimer’s disease. *PNAS* **109**(22), 8740–5. 10.1073/pnas.1206171109 (2012). 22592800 10.1073/pnas.1206171109 PMC3365221

[66] Klupp, E. et al. In alzheimer’s disease, hypometabolism in low-amyloid brain regions may be a functional consequence of pathologies in connected brain regions. *Brain Connect.* **4**(5), 371–83. 10.1089/brain.2013.0212 (2014). 24870443 10.1089/brain.2013.0212

[67] Schultz, A. P. et al. Phases of hyperconnectivity and hypoconnectivity in the default mode and salience networks track with amyloid and tau in clinically normal individuals. *J. Neurosci.* **37**(16), 4323–4331. 10.1523/JNEUROSCI.3263-16.2017 (2017). 28314821 10.1523/JNEUROSCI.3263-16.2017 PMC5413178

[68] Valderhaug, V. D. et al. Early functional changes associated with alpha-synuclein proteinopathy in engineered human neural networks. *Am. J. Physiol. Cell Physiol.* **320**, C1141-52. 10.1152/ajpcell.00413.2020 (2021). 33950697 10.1152/ajpcell.00413.2020

[69] Fiskum, V., Winter-Hjelm, N., Christiansen, N., Sandvig, A. & Sandvig, I. Als patient-derived motor neuron networks exhibit microscale dysfunction and mesoscale compensation rendering them highly vulnerable to perturbation. *bioRxiv*[SPACE]10.1101/2024.01.04.574167 (2024).

[70] Faust, T. E., Gunner, G. & Schafer, D. P. Mechanisms governing activity-dependent synaptic pruning in the developing mammalian cns. *Nat. Rev. Neurosci.* **22**, 657–673. 10.1038/s41583-021-00507-y (2021). 10.1038/s41583-021-00507-y PMC8541743 34545240

[71] van Niekerk, E. A. et al. Methods for culturing adult cns neurons reveal a cns conditioning effect. *Cell Rep. Methods* **2**(7), 100255. 10.1016/j.crmeth.2022.100255 (2022). 35880023 10.1016/j.crmeth.2022.100255 PMC9308166

[72] Evans, M. S., Collings, M. A. & Brewer, G. J. Electrophysiology of embryonic, adult and aged rat hippocampal neurons in serum-free culture. *J. Neurosci. Methods* **79**(1), 37–46. 10.1016/s0165-0270(97)00159-3 (1998). 9531458 10.1016/s0165-0270(97)00159-3

[73] Varghese, K. et al. Regeneration and characterization of adult mouse hippocampal neurons in a defined in vitro system. *J. Neurosci. Methods* **177**(1), 51–9. 10.1016/j.jneumeth.2008.09.022 (2009). 18955083 10.1016/j.jneumeth.2008.09.022

[74] Nilssen, E. S. et al. Inhibitory connectivity dominates the fan cell network in layer ii of lateral entorhinal cortex. *J. Neurosci.* **38**(45), 9712–27. 10.1523/jneurosci.1290-18.2018 (2018). 30249791 10.1523/JNEUROSCI.1290-18.2018 PMC6595991

[75] Valeeva, G. et al. Emergence of coordinated activity in the developing entorhinal–hippocampal network. *Cereb. Cortex* **29**(2), 906–920. 10.1093/cercor/bhy309 (2018). 10.1093/cercor/bhy309 PMC6319314 30535003

[76] Griguoli, M. & Cherubini, E. Early correlated network activity in the hippocampus: Its putative role in shaping neuronal circuits. *Front. Cell. Neurosci.* **11**, 255. 10.3389/fncel.2017.00255 (2017). 10.3389/fncel.2017.00255 PMC5572250 28878628

[77] Canto, C. B. & Witter, M. P. Cellular properties of principal neurons in the rat entorhinal cortex. I. The lateral entorhinal cortex. *Hippocampus* **22**(6), 1256–76. 10.1002/hipo.20997 (2012). 22162008 10.1002/hipo.20997

[78] Shao, L.-R. & Dudek, F. E. Enhanced burst discharges in the ca1 area of the immature versus adult hippocampus: patterns and cellular mechanisms. *J. Neurophysiol.* **128**(6), 1566–1577. 10.1152/jn.00327.2022 (2022). 36382903 10.1152/jn.00327.2022 PMC9744639

[79] Nedaei, H. et al. The calcium-free form of atorvastatin inhibits amyloid-42) aggregation in vitro. *J. Biol. Chem.* **298**(3), 101662. 10.1016/j.jbc.2022.101662 (2022). 35104501 10.1016/j.jbc.2022.101662 PMC8898965

[80] Percie du Sert, N. et al. The arrive guidelines 2.0: Updated guidelines for reporting animal research. *PLoS Biol.* **18**(7), e3000410. 10.1371/journal.pbio.3000410 (2020). 32663219 10.1371/journal.pbio.3000410 PMC7360023

[81] Radde, R. et al. Abeta42-driven cerebral amyloidosis in transgenic mice reveals early and robust pathology. *EMBO Rep.* **7**(9), 940–6. 10.1038/sj.embor.7400784 (2006). 16906128 10.1038/sj.embor.7400784 PMC1559665

[82] Leon, W. C. et al. A novel transgenic rat model with a full alzheimer’s-like amyloid pathology displays pre-plaque intracellular amyloid-beta-associated cognitive impairment. *J. Alzheimers Dis.* **20**(1), 113–26. 10.3233/jad-2010-1349 (2010). 20164597 10.3233/JAD-2010-1349

[83] Heggland, I., Kvello, P. & Witter, M. P. Electrophysiological characterization of networks and single cells in the hippocampal region of a transgenic rat model of alzheimer’s disease. *eNeuro*[SPACE]10.1523/eneuro.0448-17.2019 (2019). 30809590 10.1523/ENEURO.0448-17.2019 PMC6390198

[84] Richter, K. N. et al. Glyoxal as an alternative fixative to formaldehyde in immunostaining and super-resolution microscopy. *EMBO J.* **37**, 139–59. 10.15252/embj.201695709 (2017). 29146773 10.15252/embj.201695709 PMC5753035

[85] Lansey, J. C. Beautiful and distinguishable line colors + colormap, (2022).

[86] Brewer, C. A., Hatchard, G. W. & Harrower, M. A. Colorbrewer in print: A catalog of color schemes for maps. *Cartogr. Geogr. Inf. Sci.* **30**(1), 5–32. 10.1559/152304003100010929 (2003).

[87] Maccione, A. et al. A novel algorithm for precise identification of spikes in extracellularly recorded neuronal signals. *J. Neurosci. Methods* **177**(1), 241–9. 10.1016/j.jneumeth.2008.09.026 (2009). 18957306 10.1016/j.jneumeth.2008.09.026

[88] Kraus, B. Spike raster plot, (2022).

[89] Bologna, L. L. et al. Investigating neuronal activity by spycode multi-channel data analyzer. *Neural Netw.* **23**(6), 685–97. 10.1016/j.neunet.2010.05.002 (2010). 20554151 10.1016/j.neunet.2010.05.002

[90] Wang, X.-J. Pacemaker neurons for the theta rhythm and their synchronization in the septohippocampal reciprocal loop. *J. Neurophysiol.* **87**(2), 889–900. 10.1152/jn.00135.2001 (2002). 11826054 10.1152/jn.00135.2001

[91] Blondel, V. D., Guillaume, J.-L., Lambiotte, R. & Lefebvre, E. Fast unfolding of communities in large networks. *J. Stat. Mech. Theory Exp.* **2008**, P10008. 10.1088/1742-5468/2008/10/P10008 (2008).

[92] Rubinov, M. & Sporns, O. Complex network measures of brain connectivity: uses and interpretations. *Neuroimage* **52**(3), 1059–69. 10.1016/j.neuroimage.2009.10.003 (2010). 19819337 10.1016/j.neuroimage.2009.10.003

[93] Wagenaar, D. A. & Potter, S. M. Real-time multi-channel stimulus artifact suppression by local curve fitting. *J. Neurosci. Methods* **120**(2), 113–20. 10.1016/s0165-0270(02)00149-8 (2002). 12385761 10.1016/s0165-0270(02)00149-8

